# Neural Biomarkers for Dyslexia, ADHD, and ADD in the Auditory Cortex of Children

**DOI:** 10.3389/fnins.2016.00324

**Published:** 2016-07-15

**Authors:** Bettina Serrallach, Christine Groß, Valdis Bernhofs, Dorte Engelmann, Jan Benner, Nadine Gündert, Maria Blatow, Martina Wengenroth, Angelika Seitz, Monika Brunner, Stefan Seither, Richard Parncutt, Peter Schneider, Annemarie Seither-Preisler

**Affiliations:** ^1^Department of Neurology, Section of Biomagnetism, University Hospital HeidelbergHeidelberg, Germany; ^2^Division of Neuroradiology, University Hospital HeidelbergHeidelberg, Germany; ^3^Division of Radiology and Nuclear Medicine, Kantonsspital St. GallenSt. Gallen, Switzerland; ^4^Latvian Academy of MusicRiga, Latvia; ^5^Division of Neuroradiology, Department of Radiology, University of Basel HospitalBasel, Switzerland; ^6^Department of Neuroradiology, University Hospital LübeckLübeck, Germany; ^7^Department of Pediatric Neurology, University Hospital HeidelbergHeidelberg, Germany; ^8^Phoniatrics and Pedaudiology, University Hospital HeidelbergHeidelberg, Germany; ^9^Institute of Psychology, University of GrazGraz, Austria; ^10^BioTechMed GrazGraz, Austria; ^11^Centre for Systematic Musicology, University of GrazGraz, Austria

**Keywords:** auditory cortex, auditory evoked fields, synchronization, musical learning, developmental disorders, hemispheric asymmetries, magnetic resonance imaging, magnetencephalography

## Abstract

Dyslexia, attention deficit hyperactivity disorder (ADHD), and attention deficit disorder (ADD) show distinct clinical profiles that may include auditory and language-related impairments. Currently, an objective brain-based diagnosis of these developmental disorders is still unavailable. We investigated the neuro-auditory systems of dyslexic, ADHD, ADD, and age-matched control children (*N* = 147) using neuroimaging, magnetencephalography and psychoacoustics. All disorder subgroups exhibited an oversized left planum temporale and an abnormal interhemispheric asynchrony (10–40 ms) of the primary auditory evoked P1-response. Considering right auditory cortex morphology, bilateral P1 source waveform shapes, and auditory performance, the three disorder subgroups could be reliably differentiated with outstanding accuracies of 89–98%. We therefore for the first time provide differential biomarkers for a brain-based diagnosis of dyslexia, ADHD, and ADD. The method allowed not only allowed for clear discrimination between two subtypes of attentional disorders (ADHD and ADD), a topic controversially discussed for decades in the scientific community, but also revealed the potential for objectively identifying comorbid cases. Noteworthy, in children playing a musical instrument, after three and a half years of training the observed interhemispheric asynchronies were reduced by about 2/3, thus suggesting a strong beneficial influence of music experience on brain development. These findings might have far-reaching implications for both research and practice and enable a profound understanding of the brain-related etiology, diagnosis, and musically based therapy of common auditory-related developmental disorders and learning disabilities.

## Introduction

The auditory cortex (AC) is very widely connected with different brain regions, including subcortical, prefrontal and parietal areas, where attentional and default networks are hosted. In view of the strong interdependence of such integrated networks (Scheich et al., 2011; Rodriguez-Fornells et al., 2012) it is not surprising that central auditory processing disorders (CAPD), i.e. difficulties in recognizing and interpreting acoustic patterns that arise from dysfunction in the central nervous system, are often associated with attention (Sergeant et al., 2003), language, and literacy (Dawes et al., 2009) problems.

Dyslexia and AD(H)D belong to the most common developmental disorders in childhood and adolescence with a worldwide prevalence of each about 5–10% (Shaywitz and Shaywitz, 2005; Polanczyk et al., 2007). While in the normal population the prevalence of CAPD is 2–3% (Chermak et al., 1999), Hämäläinen et al. (2013) give an estimate that 30–50% of individuals with dyslexia are affected by auditory problems. Moreover, different studies have come to the conclusion that children with AD(H)D frequently also meet the criteria for CAPD (Gascon et al., 1986; Keith and Engineer, 1991; Cook et al., 1993; Gomez and Condon, 1999; Riccio et al., 2005; Ghanizadeh, 2009).

In dyslexia, poor discrimination of basic sound features and sequential acoustic patterns is held to lead to suboptimal speech representation, which constrains the development of phonological representations (Bishop et al., 1999), and hence of reading and spelling skills (Hämäläinen et al., 2013). Neuroscientific studies have provided convincing evidence for a variety of alterations in the brains of dyslexics (Shaywitz et al., 2006; Richlan et al., 2009; Gebauer et al., 2012). Longitudinal studies starting before primary school age have revealed that responses of AC to unexpected deviations in basic sound features were correlated to phonological processing and letter-naming skills at the kindergarten-age (Hämäläinen et al., 2015) and to word reading fluency at the primary school age (van Zuijen et al., 2013). In preschoolers at risk for dyslexia the amplitudes of the mismatch negativity (MMN) were found to be smaller for frequency deviants (Maurer et al., 2003) as well as vowel, vowel-duration, consonant, and intensity deviants (Lovio et al., 2010). In the Finnish “Jyväskylä Longitudinal Study of Dyslexia” 200 children, half at risk for developmental dyslexia indexed by family risk factors, were assessed from birth to puberty. Characteristics of the MMN measured at 3–5 days of age already demonstrated a significant predictive correlation to reading skills at school age (for a review see Lyytinen et al., 2015).

Similarly, ADHD involves not only problems of inattention, hyperactivity, and impulsivity, but also a core deficit in auditory, visual and motor timing (Barkley et al., 2001; Smith et al., 2002; McInerney and Kerns, 2003; Falter and Noreika, 2011; Noreika et al., 2013; Lesiuk, 2014), with timeframes ranging from milliseconds to minutes or even longer. The most frequent impairments were found in sensorimotor synchronization, duration discrimination, and time-interval reproduction (Noreika et al., 2013). Moreover, there is growing evidence for an association between perceptual timing deficits and behavioral measures of impulsiveness and inattention (Noreika et al., 2013).

One influential attentional framework was developed by Posner and Petersen (1990) and Petersen and Posner (2012) and includes three neural systems, namely the alerting network, the orienting network, and the executive network, which are linked to different attentional functions. The alerting network is associated with the brainstem arousal system along with sustained vigilance, and is strongly lateralized to the right hemisphere. The orienting network is related to parietal, frontal, and posterior brain regions and enables the favoritism of sensory input by choosing the modality or location. The executive network, which is linked to top-down regulation, is related to sustained attention, verbal and nonverbal working memory, response inhibition, and emotional/motivational self-control (Barkley, 1997). It is located in the midline frontal/anterior cingulate cortex (Posner and Petersen, 1990; Petersen and Posner, 2012). Konrad et al. (2006) provided evidence that children with ADHD show altered brain recruitments for all three attentional networks. We recently found that children with AD(H)D are also characterized by atypical hemispheric asymmetries in AC structure and function that were correlated to the severity of ADHD symptoms (Seither-Preisler et al., 2014), thus suggesting a close relationship between auditory and attentional performance. In principle, common impairments in auditory and attentional functions may be explained by a bottom-up approach, where poor auditory processing captures more attentional resources or — vice versa — by a top-down approach, where poor attention has a negative effect on auditory performance (Dawes and Bishop, 2009). It was one aim of this study to contribute to the clarification of this interdependence.

In the course of AC development from early infancy to adolescence, auditory evoked responses become faster and peaks get sharper, which signifies an increase in neural efficiency (Ponton et al., 2002; Sharma et al., 2005, 2014; Seither-Preisler et al., 2014; Dehaene-Lambertz and Spelke, 2015). There is evidence that this process is accelerated in young musicians, thus underlining the beneficial influence of musical activities on the neural foundations of auditory perception, attention, literacy (Moreno et al., 2009; Kraus and Chandrasekaran, 2010; Seither-Preisler et al., 2014; Flaugnacco et al., 2015; Tierney et al., 2015) and other cognitive functions (Schellenberg, 2004; Hyde et al., 2009). It was therefore a further aim of this study to test whether musical training could counteract developmental delays of AC on a long-term scale.

To date, AD(H)D is merely diagnosed on the basis of observable behavior, which may lead to considerable variability among informants, cultures, and countries (Chermak et al., 1999). Based on a literature review and own findings, Snyder et al. (2008) reported that accuracies of behavioral classification scales lie between 47 and 79%, which clearly affects their clinical validity. Similarly, Tripp et al. (2006) stated that overall accuracy of classifications does not exceed 76% for individual or combined rating scales of ADHD. The authors therefore suggested a multiple gating approach to behavioral ADHD assessment, which however is time-consuming and cost-intensive. In a review based on 161 randomized controlled studies, Greenhill et al. (2002) reported that due to medication improvements in AD(H)D symptoms occurred in 65–75%. This however means that in about one third of patients medical treatment was not effective, presumably due to the relatively low diagnostic validities of conventional behavioral measures.

In view of these findings, there is an urgent need for new approaches to an objective brain-based diagnosis of the developmental state of the auditory system and appropriate musically-based therapeutic strategies that may be applied by neurologists, psychologists, pediatricians, psychiatrists, and music-therapists. It was therefore the central goal of this study to clarify, whether a specified neuro-auditory profile could provide such a reliable and objective tool to diagnose auditory-related developmental disorders in general, and to differentiate between dyslexia, ADHD and ADD, in particular.

## Materials and methods

### Subjects and procedure

We measured the neuro-auditory profiles of 147 children in a cross-sectional design by means of structural MRI, MEG, and psychoacoustic tests. Participants belonged to one of four groups, namely dyslexics (*N* = 37, 26 male; mean ± SD age: 10.7 ± 1.8 years), subjects with ADHD (*N* = 37, 32 male; mean ± SD age: 10.8 ± 1.9 years), subjects with ADD (*N* = 36, 25 male; mean ± SD age: 11.0 ± 2.6 years), and normal controls (*N* = 37, 20 male; mean ± SD age: 11.0 ± 1.3 years).

All tested groups were matched as closely as possible with regard to potentially relevant background variables (Table 1). The four groups did not significantly differ in age (*F*_(3, 143)_ = 0.26, n.s.) and self-indicated handedness ($\chi_{\left(3\right)}^{2}$ = 1.7 n.s.). However, among the three disorder groups the proportion of males was considerably higher, which is consistent with the literature about the higher prevalence of AD(H)D and dyslexia in boys (Yoshimasu et al., 2010). This was not regarded as a problem, since in an earlier study we could exclude the possibility that a gender bias is responsible for neuroanatomical and functional specificities in the AC of children with ADHD and ADD (Seither-Preisler et al., 2014). All four groups were characterized by IQs (CFT20-R; Weiß, 2008) in the normal to high range. The slightly better performance of the control group (*F*_(3, 143)_ = 3.0, *p* = 0.032) may be due to a relatively higher sustained attention, which is not only impaired in ADHD and ADD, but may also be affected to some extent in dyslexia (Marzocchi et al., 2009).

**Table 1:** **Description of participants**.

	**Controls**	**Dyslexics**	**ADHD**	**ADD**	**Comorbid cases**
Number of subjects	37	37	37	36	15
Gender	M: 20	M: 26	M: 32	M: 25	M: 11
	F: 17	F: 11	F: 5	F: 11	F: 4
Handedness	R: 33	R: 34	R: 31	R: 30	R:12
	L: 4	L: 3	L: 6	L: 6	L:3
Age in years	11.0 ± 1.3	10.7 ± 1.8	10.8 ± 1.9	11.0 ± 2.6	10.1 ± 2.0
IQ	116 ± 11	108 ± 10	112 ± 14	107 ± 11	111 ± 15

*Group-specific means ± SD for age and IQ (CFT20-R; Weiß, 2008) and distributions of gender and self-indicated handedness. Comorbid cases are not included in the statistical analyses, but displayed in **Figures 2C,D***.

All subjects with disorders were officially diagnosed either by a child psychiatrist, a psychologist, or a pediatrician. In case of attentional problems, written diagnoses were obtained and subjects with the classifications F 90.0/F90.1 (ADHD) or F 98.8 (ADD) according to the International Statistical Classification of Diseases and Related Health Problems, 10th Revision (ICD-10) were included in the study. However, accuracies of behavioral classification scales lie between 47 and 79%, which clearly affects their clinical validity (Tripp et al., 2006; Snyder et al., 2008). This uncertainty is also reflected in the history of the classification schemes “Diagnostic and Statistical Manual of Mental Disorders (DSM)” of the American Psychiatric Association and the ICD system used in Europe. Since their introduction, both schemes have updated the criteria for a diagnosis of ADHD and ADD for several times in an inconsistent way, thereby contributing to a neglect of potential differences in clinical samples (Chermak et al., 1999).

In order to improve diagnostic accuracies for our sample, the original classifications were re-validated on the basis of two criteria. Informal interviews were conducted with the responsible experts to clarify if potential comorbidities had arisen after the initial diagnoses. In addition, parents had to fill out the “Parents assessment sheet for hyperactive disorder” (FBB-HKS), which is part of the “Diagnostic System for Psychiatric Disorders in Children and Adolescents” (DISYPS-KJ; Döpfner and Lehmkuhl, 2000) and contains the ICD-10 and DSM-IV criteria. In the FBB-HKS the severity of the three symptom clusters inattention, hyperactivity and impulsivity are quantified separately. If according to the age-related DISYPS reference sample the percentile rank of subjects was ≥90 on the scale “inattentiveness,” but lower on the scales “hyperactivity” and “impulsivity,” this was considered as evidence for ADD. If the percentile rank was also ≥90 either on the scale “hyperactivity,” “impulsivity” or both, this was interpreted as evidence for ADHD. Only in case of consistency between the original diagnoses and the additionally introduced criteria, subjects were included in the somewhat smaller “revalidated sample” (dyslexics: *N* = 32; ADHD: *N* = 25; ADD: *N* = 22); moreover, only controls with DISYPS-values outside the critical ranges were included (*N* = 30). All discriminant analyses were calculated as well for the original as for the more strictly defined revalidated sample.

Dyslexics were diagnosed according to the Pediatric Neurology standards of the University Hospital Heidelberg, using ELFE (Lenhard and Schneider, 2006) for reading comprehension, HSP 1–10 (May, 2012) to assess spelling skills, and H-LAD to assess phoneme discrimination (Brunner et al., 2008).

Children with an IQ below 80, as measured with the CFT20-R (Weiß, 2008), or any known comorbidities (dyscalculia, autism, epilepsy etc.) were excluded from the main study. However, an additional group of 15 children, known to be comorbid with regard to dyslexia, ADHD or ADD, was used for visual inspection of potential neurological similarities with the three unequivocally diagnosed disorder subgroups (**Figure 2C**). According to the selection criteria for our original sample, where such cases were excluded in advance as far as possible (and finally excluded in the revalidated sample), this number is lower than in the normal population (primary diagnosis “dyslexia”: 21% ADHD; primary diagnosis “ADHD”: 31% dyslexics; see Pauc, 2005).

The 147 participants of the cross-sectional study were part of a larger longitudinal project addressing the effects of musical practice on the brain and cognition from the primary school age to adolescence [“AMseL: Audio- and Neuroplasticity of Musical Learning I + II,” which is part of the accompanying research on the cultural education program “An Instrument for Every Child (JeKi)” supported by the German Federal Ministry of Education and Research].

For a sub-group of 109 children, who are all also included in the cross-sectional sample, neurological data were available for longitudinal comparisons over a time-span of 3.6 years (*SD* = 0.45). The long-term sample included 79 control children without developmental disorders (40 M, 39 F; 74 right-handers, 5 left-handers) who also participated in a larger comprehensive longitudinal study on potential benefits of musical training on brain and behavior, starting in 2009. In that study, as yet the 3rd measurement timepoint has been completed and a 4th and 5th follow-up measurement will be carried out until 2019. The cross-sectional data of this study basically correspond to the 2nd measurement timepoint. The age of the longitudinal control children was 8.5 ± 0.7 years at MTP1 and 12.2 ± 0.8 years at MTP2. The children of the control group (musicians and non-musicians) and the ADHD and ADD groups (non-musicians only) were also included in a first longitudinal comparison addressing the development of the auditory system over a shorter time span of about 1 year (Seither-Preisler et al., 2014). We found that musically trained controls revealed signs of a higher neural efficiency in AC than their musically untrained peers, while the musically untrained AD(H)D children showed a relatively lower neural efficiency. This led to the hypothesis that musical training may have a positive effect on CAPD and related developmental disorders, which however could not be directly tested due to an initial lack of musically trained AD(H)D children. Meanwhile, it was possible to recruit a sufficient number of children with dyslexia, ADHD, and ADD (pooled disorder group), who also played a musical instrument. This enabled us to directly study potential neurological long-term benefits of musical training on children with developmental disorders over a period of 3.5 years.

For the assessment of musical expertise an index of cumulative musical practice (I_MP_) was used as a classification criterion. The I_MP_ was defined as the product of the number of years of formal music education and the amount of hours per week spent practicing a musical instrument. Similarly to the cross-sectional sample, the longitudinal sub-sample was divided into (a) “control group” vs. “pooled disorder group” (all children with dyslexia, ADHD or ADD) and (b) according to the cumulative amount of musical practice at the 2nd measurement timepoint (MTP2; mean age: 12 years) into “musicians” (I_MP_ > 4) vs. “non-musicians” (I_MP_ ≤ 4). The control group comprised 79 participants (45 musicians, 34 non-musicians) and the disorder group comprised 30 participants (11 musicians, 19 non-musicians). Noteworthy, such children were very rare (less than 5%) and had to be recruited all over Germany and Switzerland over almost 1 year.

For three children (one musician and one non-musician with ADD, one musician with ADHD) the MRI or MEG data were not of sufficient quality for further processing. Accordingly, these children were only included in the analyses of psychoacoustic data and parts of the neurological analyses.

The local research ethics committee of the Medical Faculty of the Ruprecht Karls University Heidelberg approved all experimental procedures in accordance to the Helsinki declaration. Parents provided informed consent in written form and subjects informed assent.

### Morphometry

A T1-weighted structural magnetic MRI (Siemens, TrioTim, 3 Tesla, software version: “syngo MR B17,” MPRAGE, 176 DICOM slices, sagittal orientation; slice thickness 1 mm, field of view: 256 × 256; matrix size 128 K (16 Bit), repetition time (TR) = 1930 ms, echo time (TE) = 3.47 ms, flip angle 15°) was performed to investigate the anatomy of AC. A standardized individual approach of three-dimensional gray matter surface reconstruction of auditory subareas (HG, PT) was applied to account for individual morphology and gyrification patterns (Schneider et al., 2002, 2005, 2009). For segmentation the Brain Voyager software QX 2.8 (Brain Innovation, B.V, Maastricht, NL) was used. All brain images got adjusted in contrast and in brightness, were precisely corrected for inhomogeneity and rotated in direction of the antero-posterior commissural line. Normalization in stereotactic space (Talairach and Tournoux, 1988) was carried out in order to compute group averaged AC surfaces. The superior temporal plane, including HG, the anterior superior temporal gyrus (aSTG) and PT, was segmented into sagittal MRI slices along the Sylvian fissure using the standard definition of the landmarks of AC and approved additional criteria: The first complete Heschl's sulcus (cHS) with a large mediolateral extent (>97%) and pronounced depth was used as the posterior boundary and the crescent-shaped first transverse sulcus (FTS) as the anterior boundary of HG, thereby dividing AC into two parts: (1) an anterior stream including HG and aSTG and (2) a posterior stream including PT. HG was separated from aSTG by an anterior borderline with *y* = 0 (Schneider et al., 2005; Wengenroth et al., 2010; Seither-Preisler et al., 2014). The range of the included image gray values was calculated individually. A box was marked around left and right AC to generate intensity histograms of these areas. The “gray value inclusion range,” which was used for surface reconstruction and morphometry, was defined on the basis of two criteria: (1) the value of the gray matter peak multiplied by the factor 0.28, which characterizes an appropriate cutoff value to separate liquor from gray matter tissue, (2) the saddle point between gray and white matter peaks. The gray and white value voxels embedded in this inclusion range were marked and used for 3D reconstruction; for morphometry only gray matter values were used.

### Magnetoencephalography

A Neuromag-122 whole-head MEG system was used to measure and analyze the response of AC to acoustic stimuli. First the locations of four head position coils together with a set of 35 surface points including nasion and two pre-auricular points were digitized in a preparation room. Due to automatic artifact correction provided by the Brain Electromagnetic Source Analysis (BESA Software GmbH, Version 6.0; Graefelfing) it was not necessary to acquire EOGs. Then subjects were led to the magnetically shielded room, where the MEG measurements took place. They were requested to sit relaxed under the dewar of the MEG system. Foam ear pieces (Ethymotic ER3) were connected via 90 cm plastic tubes (diameter 3 mm) to small shielded transducers that were fixed in boxes next to the subject's chair. At the beginning of the MEG recordings the head position inside the dewar was determined. The loudness of the stimulation was adjusted to an equal subjective loudness (50 dB nSL) as determined by a Brüel and Kjaer artificial ear (type 4152). Stimuli were presented binaurally.

Children were instructed to listen passively to the presented sounds over a measurement-duration of about 15 min. They were allowed to watch a silent movie in order to keep them seated and calm and to reduce potential artifacts. The stimuli were presented in pseudo-randomized order and consisted of seven different instrumental sounds (piano, trumpet, flute, plucked violin, bass clarinet, and timpani) and four synthetically generated harmonic complex tones, each with a duration of 500 ms and a pseudo-randomized interstimulus interval between 400 and 500 ms. Each of the 11 sounds was presented 100 times, while auditory evoked fields were recorded. This high repetition rate guaranteed a good signal-to-noise-ratio as a necessary predisposition for robust source modeling and reliable source waveform analyses.

The auditory evoked fields were recorded with a bandpass filter of 0.00 (DC)−330 Hz and a sampling rate of 1000 Hz. Prior to averaging, data were automatically inspected to exclude external artifacts by using the BESA Research Event-Related Fields (ERF) module. By applying the automatic Artifact Scan tool, on average about 3–7 noisy (bad) channels were excluded and about 10% of all epochs exceeding a gradient of 600 fT/cm×s and amplitudes either exceeding 3000 fT/cm or falling below 100 fT/cm were rejected from further analysis. Thereby, the major part of endogenous artifacts, like eye blinks, eye movements, cardiac activity, face movements, and muscle tensions could be accounted for. Due to the extremely high signal-noise-ratio (noise reduction factor of 33.2 when averaging over 1100 epochs) and systematic pseudo-randomization of the interstimulus intervals (see above), the influence of potential remaining artifacts was negligible. The efficiency of the procedure has also been demonstrated in a pilot study, where two additional EOG electrodes and one ECG electrode were used during the recordings and where continuous data were subsequently corrected manually for vertical and horizontal eye movements and ECG artifacts (Ille et al., 2002). A baseline-amplitude calculated over the 100 ms-interval before the onset of the tones was subtracted from the data. The responses of each subject were first collapsed into a grand average (1100 artifact-free epochs) in a 100 ms pre-stimulus to 400 ms post-stimulus time window. Based on a spherical head model (Hämäläinen and Sarvas, 1987; Sarvas, 1987), spatio-temporal source modeling was performed for the P1 response complex by using one regional source in each hemisphere. The fitting intervals were individually adjusted by using the lower and upper half-side lobe around the P1 peak and setting the dipole orientation to its maximum. The linear source showing the maximal amplitude was orientated toward the vertex and used for further analyses of P1 latency, width, and amplitude. The high temporal accuracy of P1 peak latency is independent of the exact source location in AC (Wengenroth et al., 2014). Apart from P1 peak latency, amplitude and width (distance between half-side lobes), absolute P1 asynchrony |P1(Peak){right − left}| was calculated as an indicator of the synchronization between the left and right AC (Seither-Preisler et al., 2014). Furthermore, Δ-values [(R−L)/(R+L)] indicating the relative predominance of the right (positive) or left (negative) hemisphere: “ΔL (asymmetry of P1 latency),” “ΔW (asymmetry of P1 width),” “ΔA (asymmetry of P1 amplitude)” were determined. The later N1 response was not included in the analysis, because it is still weak and considerably decelerated in elementary school children and develops until the end of puberty (Ponton et al., 2002).

### Auditory tests

For the audiometric and psychoacoustic tests stimuli were presented binaurally with a Hammerfall DSP Multiface System and closed dynamic headphones (Sennheiser HDA 200) designed for high-quality auditory testing. These headphones provide a passive attenuation of about 30 dB within the frequency range of the used stimuli. Stimulus intensity was adjusted to 65 dB SPL using a Brüel and Kjaer artificial ear, type 4152. The order of the behavioral tasks and neurological measurements was counterbalanced to avoid sequence effects. The total measurement time of the behavioral tests was about 3 h including three to four breaks (each about 10–15 min) between the different tasks.

#### Dinosaur tests

In order to test basic sound discrimination abilities, the “Dinosaur” threshold estimation program (Sutcliffe and Bishop, 2005; Huss et al., 2011) was used. Difference limes are measured for tone frequency (“low vs. high”), intensity (“soft vs. loud”), onset ramp (“mellow vs. sharp”), and duration (“short vs. long”). In an alternative forced-choice paradigm, reference and test tones (sinusoids) separated by an interstimulus interval of 500 ms are presented. Participants are introduced to cartoon animals and are told that each one would make a sound. They are asked to decide per mouse-click, which of the presented tones sounds higher, softer, sharper, or longer. Immediate feedback is given by the computer program throughout the experiment. An adaptive staircase procedure (after 2 reversals, a 2-up 1-down procedure changes into 3-up 1-down) is used to adapt stimulus difficulty to the participant's previous answer. The threshold is based on the point of 75% correct responses for the last four reversals. As a consequence, the number of completed trials varies slightly among participants, with a maximum of 40 possible trials. In the frequency subtest the standard is a 500 Hz pure tone and the frequency difference between the tones varies randomly up to a maximum of two semitones. In the intensity subtest the standard is fixed at 65 dB SPL, while the test tones vary from 45 to 65 dB SPL. In the onset ramp subtest the standard has a 15 ms linear rise time, a 735 ms steady segment, and a 50 ms linear fall time, while the rise times of the test tones vary logarithmically up to 300 ms. In the duration subtest the standard has a duration of 400 ms and the comparison tones are varied logarithmically from 400 to 600 ms.

#### Metric test

A short version of the Metric Test (Sutcliffe and Bishop, 2005; Huss et al., 2011) was used to test the ability to detect temporal anisochronies in acoustic sequences induced by variations in musical meter. The metrical arrangements consist of series of notes in 4/4 or 3/4 time with a pitch of 392 Hz (G) and an underlying pulse rate of 500 ms. Musical accent is given to the first, second or third note in a bar by increasing the intensity of the relevant note by 5 dB. Among the 24 presented trials half of the standard and comparison sequences are identical and half are slightly different. A change in metrical structure is either caused by adding 100 ms (short duration change) or 166 ms (long duration change) to the accented note, with both variants occurring at the same rate. Trials are presented in pseudo-randomized order, with each trial occurring twice. Participants are instructed to decide per mouse-click whether the presented arrangements are the same or different. Immediate feedback is given during the entire experiment by the computer program.

#### Phoneme discrimination test (H-LAD)

The Heidelberger Lautdifferenzierungstest (H-LAD, Brunner et al., 2008) is a test of auditory perception and phonological awareness in dyslexia, which consists of three subtests that are presented via PC:

1. Phoneme discrimination: Pairs of words and non-words, which differ by a single phonemic feature; the targets comprise different voice onset times (e.g., /ba/ vs. /pa/) or formant transitions (e.g., /da/ vs. /ga/). Participants have to decide, whether the items sound equal or different.
2. Verbal repetition: Subjects have to repeat the word or non-word pairs directly after presentation.
3. Analysis of consonant clusters with voiced and voiceless stop sounds at the initial position of a word: Subjects have to tell, which is the first and which the second phoneme of the presented word.

Each subtest may be evaluated separately; a total score is obtained by summing up all correct answers (maximum: 62). Normative data (percentile ranks and *T*-values) are available for children of grade 1, 2, 3, and 4.

#### Intermediate measures of music audiation (IMMA)

The IMMA test by Gordon (1986) measures musical aptitude by assessing the ability to internalize musical structures and to find rhythmic or melodic (tonal) variations in sequentially presented patterns. 40 pairs of tone sequences and 40 pairs of rhythms are presented via CD and the children's task is to decide whether they are equal or different by marking two identical or different smilies on an answer sheet. Accordingly, the rhythmic and melodic subtest scores can reach a maximum of 40 correct answers each.

#### Auditory ambiguity test (AAT)

A short version of the AAT (Seither-Preisler et al., 2007) was used to test the individual tendency to perceive harmonic sounds either in terms of concrete spectral patterns (timbre) or in terms of an abstract missing fundamental frequency. The relative predominance of these two aspects of subjective pitch perception was considered as relevant, because a previous study had shown a corresponding hemispheric specialization, with spectral timbre being represented in the right and fundamental pitch being represented in the left Heschl's Gyrus, respectively (Schneider et al., 2005). The short version of the test consists of 40 ambiguous tone sequences in which a rise in the spectrum is associated with a falling missing fundamental and vice versa. Each tone has a linearly ascending and descending ramp of 10 ms and a plateau of 480 ms. The time interval between tones is 500 ms and the time interval between two successive trials is 4000 ms. Sequences are presented in pseudorandomized order. Participants have to assess in a two-alternative forced-choice paradigm, whether the subjective pitch of a tone sequence goes up or down. The score that can be achieved varies from 0% (only spectrally based responses) to 100% (only responses based on the missing fundamental frequency). The tones of a pair have one of the following spectral profiles: (a) low-spectrum tone: 2nd–4th harmonic, high-spectrum tone: 5th–10th harmonic; (b) low-spectrum tone: 3rd–6th harmonic, high-spectrum tone: 7th–14th harmonic; (c) low-spectrum tone: 4th–8th harmonic, high spectrum tone: 9th–18th harmonic.

### Statistical analyses

For correlational analyses we used Pearson's coefficients, if according to the Kolmogorov-Smirnov Test both tested variables were normally distributed. Otherwise, the non-parametric Spearman's Rho was used.

For the analysis of neurological specificities, three-way ANOVAs were calculated for the independent variables “Disorder” (controls, dyslexics, ADHD, ADD), “Musical expertise” (musicians, non-musicians), and “Hemisphere” (R, L). For the MRI-data “HG volume,” “PT volume,” and “HG/PT-ratio” and for the MEG data “P1 latency,” “P1 width,” and “P1 amplitude” were considered as dependent variables. Moreover, measures of functional lateralization were introduced and considered in corresponding two-way ANOVAs. These comprised “Absolute P1 asynchrony” and the relative asymmetry values “ΔL,” “ΔW,” and “ΔA.”

Likewise, performance in each of the psychoacoustic tests was analyzed in two-way ANOVAs with the dependent variables “Difference limens for frequency,” “intensity,” “onset ramp,” “duration,” “meter,” “rhythm,” “melody,” as well as “Subjective pitch perception.”

In case of a significant main effect “Disorder” and homogeneous error variances (as indicated by the Levene-Test), *post-hoc* comparisons between controls, dyslexics, ADHD-children, and ADD-children were performed with the Tukey-Test (including a Bonferroni correction for multiple comparisons). Otherwise, the Tamhane Test was used. In case of significant interactions, the mean values of interest were compared with the Tukey-HSD.

For the supplementary analysis of longitudinal data on the effects of musical practice a 2-way ANOVA was performed on the independent variables “Disorder” (controls, pooled disordered) and “Musical expertise” (non-musicians, musicians) and the dependent variable “Absolute P1 asynchrony.”

In addition, discriminant analyses were performed to investigate how well different neural parameters segregate (a) the control group from the pooled disorder group and (b) the three disorder subgroups (dyslexia, ADHD, ADD). The considered predictor variables were (1) MRI-based morphology of AC: “HG/PT ratio right,” “HG/PT ratio left”; (2) MEG-based functional lateralization of AC: “Absolute P1-asynchrony,” “ΔL,” “ΔW,” “ΔA”; (3) Behavioral measures: (a) Auditory discrimination of frequency, intensity, onset-ramp, tone duration (Dino Test) and meter (Metric Test); (b) Musicality (rhythmic and melodic scores of IMMA); (c) Subjective pitch (AAT-score).

Sensitivity, Specificity, and Hit rate. The sensitivity “SEN” is defined as the capacity to correctly identify cases with deficit = N_correct positive_/(N_correct positive_ + N_false negative_); the specificity “SPEC” indicates the capacity to correctly identify non-deficit cases = N_correct negative_/(N_correct negative_ + N_false positive_). The hit rate indicates the proportion of correctly classified cases in general = (N_correct positive_ + N_correct negative_)/N_all cases_.

All statistical analyses were carried out with the software package IBM SPSS Statistics Version 21.0.0.0.

## Results

### Morphometric group differences

**Figure 1:**
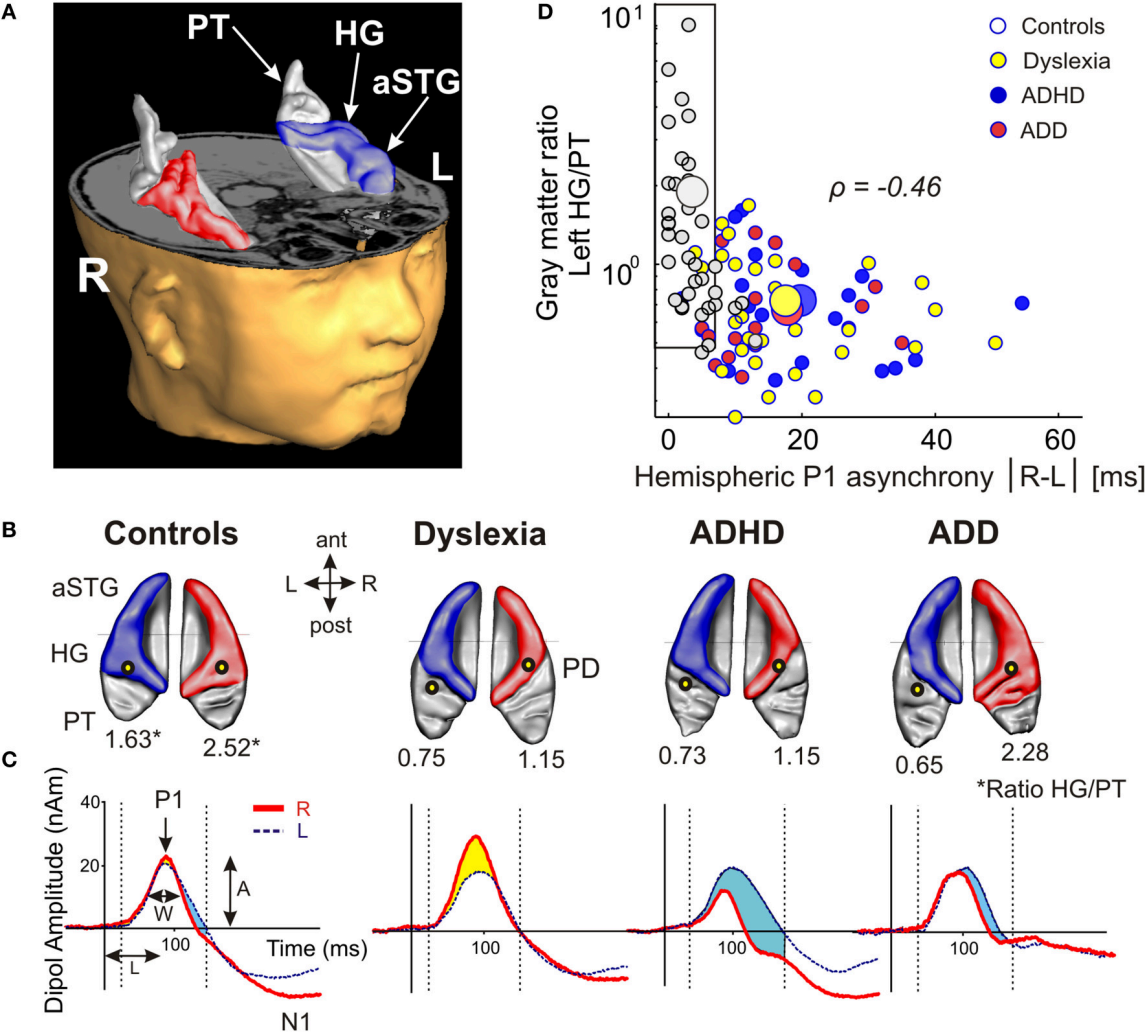
**Structural and functional auditory-related neuromarkers of dyslexia, ADHD and ADD. (A)** 3D reconstruction of an individual AC; Heschl's gyrus, its duplications and anterior superior gyrus (aSTG) are colored in blue (left hemisphere) and red (right hemisphere), respectively. The planum temporale (posterior triangular structure) and planum polare (anterior to the first transverse sulcus) are displayed in gray. **(B)** Top view of group-averaged auditory cortices (L, left; R, right, ant, anterior; post, posterior). The left hemisphere is characterized by relatively larger PTs (group average 5703 mm^3^) than the right hemisphere (3662 mm^3^). The mean ratios of HG/PT gray matter volumes (marked by asterisks) for groups and hemispheres are indicated by numbers. In the left hemisphere all disorder subgroups showed oversized PTs and downsized HGs as compared to controls, resulting in diminished left HG/PT ratios. However, only dyslexics and children with ADHD were characterized by enlarged right PTs and consequently lower right HG/PT ratios. In contrast, subjects with ADD did not show any right-hemispheric volumetric anomalies. Sources of the primary P1 responses to acoustic stimulation are projected onto the group-averaged surface meshes (yellow circles). While for controls the P1 sources localize robustly on left and right HG, all disorder subgroups show an atypical left-hemispheric focus of activation with a more posterior P1 source in PT (for Talairach coordinates see Table 3). **(C)** Group-averaged P1 source waveforms in response to the sounds of various musical instruments and artificial tones for the right (red curve) and left (blue curve) hemisphere. In contrast to controls, all three disorder subgroups demonstrated considerably different bilateral activation patterns (yellow and blue shaded areas: stronger right- and left-hemispheric activation, respectively). Usually, the P1 response was delayed on the left side, however 23% of the children showed a reversed pattern. We therefore calculated the absolute P1 asynchrony (|R–L|) as a general measure of bi-hemispheric latency divergence. Controls showed well-balanced response patterns with an average absolute P1 asynchrony of 3.7 ms, whereas all three disorder subgroups showed asynchronies that were about five times larger (ADHD: 19.4 ms; ADD: 17.5 ms; dyslexia: 16.5 ms). **(D)** Correlation between the relevant neuroanatomical and–functional measures “left HG/PT ratio” and “absolute P1 asynchrony,” which together allow an almost perfect separation of controls (gray circles) and the pooled disorder group (colored circles). Large symbols indicate mean values.

Using standard segmentation techniques (Schneider et al., 2005, 2009) the AC including Heschl's gyrus (HG), planum temporale (PT) and anterior supratemporal gyrus (aSTG) was extracted from the structural T1-weighted MR images (Figure 1A). Individual and group-specific analyses revealed remarkable differences in gyrification, size and hemispheric asymmetry with regard to the presence and type of developmental disorder (Figure 1B); for mean values, standard errors of the mean (SEM), statistical significance values and effect sizes (partial η^2^) please refer to Table 2.

**Table 2:** **ANOVA results for MRI-based gray matter volumes of Heschl's gyrus (HG), Planum temporale (PT), and HG/PT ratio in the right (R) and left (L) hemisphere (variable “Hem”)**.

	**Effect**	**Mean ± SEM**			**Significance**	***Post-hoc* Comparisons^2^**
HG volume (mm^3^)	Hem	R	4219 ± 100		n.s.	
		L	4051 ± 85			
	Dis	Cont	4597 ± 157		*F*_(3, 138)_ = 4.2,	Cont vs. Dysl: *p* < 0.01
		Dysl	3877 ± 156		*p* = 0.007,	Cont vs. ADHD: *p* < 0.01
		ADHD	3953 ± 159		partial η^2^ = 0.08	Cont vs. ADD: *p* < 0.05
		ADD	4113 ± 165			
	ME	Non	3724 ± 108		*F*_(1, 138)_ = 26.5, *p* = 8.7 × 10^−7^, partial η^2^ = 0.16	
		Mus	4545 ± 118			
	Hem × Dis	Group	R	L	*F*_(3, 138)_ = 4.8, *p* = 0.003, partial η^2^ = 0.09	R-Cont vs. R-Dysl: *p* < 0.01
		Cont	4532 ± 196	4662 ± 168		R-Dysl vs. R-ADD: *p* < 0.01
		Dysl	3795 ± 196	3959 ± 168		L-Cont vs. L-Dysl: *p* < 0.01
		ADHD	4062 ± 200	3844 ± 171		L-Cont vs. L-ADHD: *p* < 0.01
		ADD	4487 ± 207	3739 ± 177		L-Cont vs. L-ADD: *p* < 0.01
	Hem × ME	Non	3650 ± 134	3798 ± 115	*F*_(1, 138)_ = 11.0, *p* = 0.001, partial η^2^ = 0.07	R-mus vs. L-mus: *p* < 0.01
		Mus	4788 ± 147	4303 ± 126		R-non vs. R-mus: *p* < 0.01
						L-non vs. L-mus: *p* < 0.01
PT volume (mm^3^)	Hem	R	3662 ± 136		*F*_(1, 138)_ = 111.3, *p* = 1.9 × 10^−19^, partial η^2^ = 0.45	
		L	5703 ± 206			
	Dis	Cont	3407 ± 286		*F*_(3, 138)_ = 9.6, *p* = 8.9 x 10^−6^, partial η^2^ = 0.17	Cont vs. Dysl: *p* < 0.01
		ADHD	5238 ± 290			Cont vs. ADHD: *p* < 0.01
		ADD	4766 ± 300			Cont vs ADD: *p* < 0.01
		Dysl	5319 ± 285			
	ME	non	5268 ± 196		*F*_(1, 138)_ = 16.2, *p* = 9.1 × 10^−5^, partial η^2^ = 0.10	
		mus	4097 ± 214			
	Hem × Dis	Group	R	L	*F*_(3, 138)_ = 5.3, *p* = 0.002, partial η^2^ = 0.10	R-Cont vs. R-Dysl: *p* < 0.05
		Cont	2872 ± 268	3953 ± 405		R-Cont vs. R-ADHD: *p* < 0.01
		Dysl	4178 ± 267	6461 ± 403		R-ADHD vs. R-ADD: *p* < 0.05
		ADHD	4414 ± 272	6061 ± 411		L-Cont vs. L-Dysl: *p* < 0.01
		ADD	3183 ± 282	6349 ± 426		L-Cont vs. L-ADHD: *p* < 0.01
						L-Cont vs. L-ADD: *p* < 0.01
HG/PT Ratio	Hem	R	1.77 ± 0.1		*F*_(1, 138)_ = 65.0, *p* = 3.2 × 10^−13^, partial η^2^ = 0.32	
		L	0.94 ± 0.06			
	Dis	Cont	2.07 ± 0.12		*F*_(3, 138)_ = 19.2, *p* = 1.8 × 10^−10^, partial η^2^ = 0.30	Cont vs. Dysl: *p* < 0.01
		Dysl	0.95 ± 0.13			Cont vs. ADHD: *p* < 0.01
		ADHD	0.94 ± 0.13			Cont vs ADD: *p* < 0.01
		ADD	1.46 ± 0.13			
	ME	Non: 0.85 ± 0.08			*F*_(1, 138)_ = 66.5, *p* = 1.9 × 10^−13^, partial η^2^ = 0.33	
		Mus: 1.86 ± 0.09				
	Hem × Dis	Group	R	L	*F*_(3, 138)_ = 7.4, *p* = 1.3 × 10^−4^, partial η^2^ = 0.14	R-Cont vs. R-Dysl: *p* < 0.01
		Cont	2.52 ± 0.19	1.63 ± 0.13		R-Cont vs. R-ADHD: *p* < 0.01
		Dysl	1.15 ± 0.19	0.75 ± 0.13		R-ADHD vs. R-ADD: *p* < 0.01
		ADHD	1.15 ± 0.19	0.73 ± 0.14		R-Dysl vs. R-ADD: *p* < 0.01
		ADD	2.28 ± 0.20	0.65 ± 0.14		L-Cont vs. L-Dysl: *p* < 0.01
						L-Cont vs. L-ADHD: *p* < 0.01
						L-Cont vs. L-ADD: *p* < 0.01
	Hem × ME	Non	1.02 ± 0.13	0.68 ± 0.09	*F*_(1, 138)_ = 22.9, *p* = 4 × 10^−6^, partial η^2^ = 0.14	R-non vs. L-non: *p* < 0.05
		Mus	2.53 ± 0.14	1.19 ± 0.1		R-mus vs. L-mus: *p* < 0.01
						R-non vs. R-mus: *p* < 0.01
						L-non vs. L-mus: *p* < 0.01

*Group comparisons address (1) the influence of the presence and type of disorder [variable “Dis”; subgroups: “controls” (Cont), “dyslexia” (Dysl), “ADHD,” and “ADD”] and (2) the influence of musical expertise [variable “ME”; subgroups: “non-musicians” (Non) and “musicians” (Mus)]. Morphometric values: mean (mm^3^) ± standard error of mean (SEM)*.

We have shown previously for young children that such individual differences in the macroscopic and visible gross-morphology of AC are extremely stable over time and are likely to be mediated by genetic dispositions and/or by prenatal and early environmental influences (Seither-Preisler et al., 2014). They hence may be considered as stable markers of the investigated developmental disorders in childhood and adolescence.

**Table 3:** **Source locations of the primary auditory evoked P1 responses**.

		**Controls**	**Dyslexics**	**ADHD**	**ADD**
x-Coordinate	R	47.1 ± 0.9	47.3 ± 1.1	46.7 ± 0.9	47.3 ± 0.8
	L	−46.3 ± 1.1	−47.9 ± 1.1	−49.8 ± 0.8	−48.9 ± 1.1
y-Coordinate	R	−14.1 ± 1.2	−13.3 ± 1.5	−14.8 ± 1.5	−13.6 ± 1.7
	L	−15.6 ± 1.1	−23.7 ± 1.1	−25.1 ± 1.5	−25.7 ± 1.9

*x- and y-coordinate in Talairach stereotaxic space; mean values (mm) ± SEM*.

All three disorder subgroups showed systematically smaller HG and consequently enlarged PT volumes in the left hemisphere, resulting in considerably smaller left HG/PT ratios as compared to controls. The right hemisphere revealed a more specific pattern. Here, HG/PT ratios were generally higher and about the same in controls and ADD children, but significantly decreased in children with ADHD and dyslexia. With regard to gyrification patterns, dyslexics were characterized by the presence of complete posterior right HG duplications (31 out of 37 cases, see Figure 1B, marked as “PD”), which occurred at a much lower rate (<15%) in the other groups. According to earlier definitions (Schneider et al., 2005; Seither-Preisler et al., 2014), these complete posterior duplications are considered to be part of PT. Furthermore, children with ADD showed a higher incidence of right HG duplications than children with ADHD, with various types of gyrification patterns, including common stem, medial, lateral and complete HG duplications.

As expected from our previous findings (Seither-Preisler et al., 2014), the HG/PT ratios of young “musicians” were considerably higher (1.86 ± 0.09) than those of “non-musicians” (0.85 ± 0.08). Irrespective of the presence and type of disorder, the musicality-effect was lateralized and stronger in the right hemisphere (HG/PT ratios right: musicians: 2.53 ± 0.14, non-musicians: 1.02 ± 0.13; left: musicians: 1.19 ± 0.1, non-musicians: 0.68 ± 0.09), hence corroborating the predominant role of right AC for musical aptitude and learning.

### Auditory evoked fields

The morphological findings were reflected in the source locations and temporal dynamics of the auditory evoked fields in response to the sounds of musical instruments and harmonic complex tones, as measured by MEG (see Materials and Methods). We focused on the first positive response complex (P1), peaking around 60–110 ms after tone onset and being the most prominent component in children at the elementary school age.

All three disorder subgroups showed aberrant mean P1 source locations (*F*_(3, 141)_ = 11.2, *p* = 1.3 × 10^−6^, part. η^2^ = 0.19). As evident from Table 3, relative to normal controls sources were located significantly more posteriorly in the left hemisphere in children with dyslexia (*p* = 9.4 × 10^−6^), ADHD (*p* = 1.7 × 10^−5^), and ADD (*p* = 0.0001). While controls showed fairly symmetric P1 sources in left and right HG, all disorder subgroups were characterized by atypical locations on left PT. The result is graphically illustrated in Figure 1B. The group averaged residual variances for the used two-dipole model were 12.4% (±1.4 SEM) for controls, 14.5% (±1.7 SEM) for dyslexics, 17.2% (±1.6 SEM) for ADHD children, and 17.9% (±1.6 SEM) for ADD children.

Responses to acoustic stimulation were generally faster on the right side (group-averaged P1 peak latencies right: 80.7 ± 1.0 ms, left: 89.9 ± 1.2 ms), but varied substantially across groups. Controls showed well-balanced bilateral responses with an average absolute P1-asynchrony (peak latency difference) of 3.7 ± 1.6 ms. Intriguingly, such asynchronies were about five times larger in the disorder subgroups (dyslexia: 16.5 ± 1.6 ms; ADHD: 19.4 ± 1.6 ms; ADD: 17.5 ± 1.7 ms); for statistical significance values and effect sizes (partial η^2^) please refer to Table 4.

**Table 4:** **ANOVA results for MEG-based auditory evoked P1 responses in the right (R) and left (L) hemisphere**.

	**Effect**	**Mean ± SEM**			**Significance**	***Post-hoc* Comparisons^2^**
P1 latency (ms)	Hem	R	80.7 ± 1.0		*F*_(1, 137)_ = 50.7, *p* = 5.5 × 10^−11^, partial η^2^ = 0.27	
		L	89.9 ± 1.2			
	Dis	Cont	83.0 ± 1.7		n.s.	
		Dysl	86.7 ± 1.7			
		ADHD	85.7 ± 1.7			
		ADD	85.6 ± 1.9			
	ME	Non	87.8 ± 1.2		*F*_(1, 137)_ = 7.7, *p* = 6.2 × 10^−3^, partial η^2^ = 0.05	
		Mus	82.8 ± 1.3			
	Hem × Dis	Group	R	L	*F*_(3, 137)_ = 6.8, *p* = 2.5 × 10^−4^, partial η^2^ = 0.06	L-Cont vs. L-ADHD: *p* < 0.01
		Cont	81.9 ± 2.0	84.1 ± 2.3		R-Dysl vs. L-Dysl: *p* < 0.05
		Dysl	82.5 ± 2.0	91.0 ± 2.3		R-ADHD vs. L-ADHD: *p* < 0.01
		ADHD	76.7 ± 2.0	94.8 ± 2.3		R-ADD vs. L-ADD: *p* < 0.05
		ADD	81.6 ± 2.1	89.6 ± 2.5		
P1 asynchrony |R–L|	Dis	Cont	3.7 ± 1.6		*F*_(3, 137)_ = 20.3, *p* = 6.2 × 10^−11^, partial η^2^ = 0.31	Cont vs. Dysl: *p* < 0.01
		Dysl	16.5 ± 1.6			Cont vs. ADHD: *p* < 0.01
		ADHD	19.4 ± 1.6			Cont vs. ADD: *p* < 0.01
		ADD	17.5 ± 1.7			
	ME	Non	16.6 ± 1.1		*F*_(1, 137)_ = 8.0, *p* = 5.3 × 10^−3^, partial η^2^ = 0.06	
		Mus	12.0 ± 1.2			
ΔL	Dis	Cont	−0.013 ± 0.014		*F*_(3, 137)_ = 6.6, *p* = 3.9 × 10^−4^, partial η^2^ = 0.12	Cont vs. ADHD: *p* < 0.01
		Dysl	−0.045 ± 0.014			Dysl vs. ADHD: *p* < 0.05
		ADHD	−0.102 ± 0.014			ADHD vs. ADD: *p* < 0.05
		ADD	−0.049 ± 0.016			
	ME	Non	−0.047 ± 0.010		n.s.	
		Mus	−0.058 ± 0.011			
P1 width (ms)	Hem	R	43.9 ± 1.4		*F*_(1, 137)_ = 22.5, *p* = 5.3 × 10^−6^, partial η^2^ = 0.14	
		L	51.0 ± 1.3			
	Dis	Cont	46.1 ± 2.2		n.s.	
		Dysl	46.5 ± 2.2			
		ADHD	49.7 ± 2.2			
		ADD	47.3 ± 2.4			
	ME	Non	47.7 ± 1.5		n.s.	
		Mus	47.1 ± 1.7			
	Hem × Dis	Group	R	L	*F*_(3, 137)_ = 17.7, *p* = 8.8 × 10^−10^, partial η^2^ = 0.28	R-Dysl vs. R-ADHD: *p* < 0.01
		Cont	43.8 ± 2.7	48.4 ± 2.6		R-ADHD vs. R-ADD: *p* < 0.05
		Dysl	48.0 ± 2.7	45.1 ± 2.6		L-Cont vs. L-ADHD: *p* < 0.01
		ADHD	37.2 ± 2.7	62.2 ± 2.6		L-Dysl vs. L-ADHD: *p* < 0.01
		ADD	46.6 ± 2.9	48.1 ± 2.8		L-ADHD vs. L-ADD: *p* < 0.01
						R-ADHD vs. L-ADHD: *p* < 0.01
ΔW	Dis	Cont	−0.055 ± 0.031		*F*_(3, 137)_ = 16.7, *p* = 2.6 × 10^−9^, partial η^2^ = 0.27	Cont vs. ADHD: *p* < 0.01
		Dysl	+0.034 ± 0.031			Dysl vs. ADHD: *p* < 0.01
		ADHD	−0.256 ± 0.031			ADHD vs. ADD: *p* < 0.01
		ADD	−0.012 ± 0.034			
	ME	Non	−0.095 ± 0.021		n.s.	
		Mus	−0.049 ± 0.023			
P1 amplitude (nAm)	Hem	R	28.1 ± 1.2		n.s.	
		L	29.1 ± 1.1			
	Dis	Cont	34.3 ± 2.0		*F*_(3, 137)_ = 4.9, *p* = 0.003, partial η^2^ = 0.10	Cont vs. ADHD: *p* < 0.01
		Dysl	29.2 ± 2.0			
		ADHD	23.4 ± 2.0			
		ADD	27.7 ± 2.2			
	ME	Non	29.0 ± 1.4		n.s.	
		Mus	28.2 ± 1.5			
	Hem × Dis	Group	R	L	*F*_(3, 137)_ = 15.1, *p* = 1.4 × 10^−8^, partial η^2^ = 0.25	R-Cont vs. R-ADHD, *p* < 0.01
		Cont	32.4 ± 2.5	36.2 ± 2.1		R-Dysl vs. R-ADHD: *p* < 0.01
		Dysl	34.8 ± 2.4	23.5 ± 2.1		R-Dysl vs. R-ADD: *p* < 0.01
		ADHD	19.2 ± 2.5	27.5 ± 2.1		R-ADHD vs. R-ADD: *p* < 0.05
		ADD	26.1 ± 2.7	29.3 ± 2.3		L-Cont vs. L-Dysl: *p* < 0.01
						L-Cont vs. L-ADHD: *p* < 0.01
						L-Cont vs. L-ADD: *p* < 0.05
						R-ADHD vs. L-ADHD: *p* < 0.01
						R-Dysl vs. L-Dysl: *p* < 0.01
ΔA	Dis	Cont	−0.073 ± 0.037		*F*_(3, 137)_ = 20.4, *p* = 5.6 × 10^−11^, partial η^2^ = 0.31	Cont. vs. Dsyl: *p* < 0.01
		Dysl	+0.187 ± 0.036			Cont vs. ADHD: *p* < 0.05
		ADHD	−0.210 ± 0.037			Dysl vs. ADHD: *p* < 0.01
		ADD	−0.050 ± 0.040			Dysl vs. ADD: *p* < 0.01
	ME	Non	−0.072 ± 0.025		n.s.	
		Mus	−0.001 ± 0.028			

*Group comparisons address (1) the influence of the presence and type of disorder [variable “Dis”; subgroups: “controls” (Cont), “dyslexia” (Dysl), “ADHD,” and “ADD”] and (2) the influence of musical expertise [variable “ME”; subgroups: “non-musicians” (Non) and “musicians” (Mus)]. P1-parameters: (1) “P1 latency”: time point of peak value (ms), (2) “P1 width”: distance between ascending and descending half-side lobe of P1 peak (ms), (3) “P1 amplitude”: peak value (nAm: nanoamperemeter), (4) “P1 asynchrony”: absolute P1 latency difference |R–L| (ms); (5) ΔL, ΔW, and ΔA: relative asymmetries [(R-L)/(R+L)] of P1 latency, width and amplitude (positive values: right predominance, negative values: left predominance). Indicated values: mean ± SEM*.

When relating AC morphology (HG/PT ratio left) to functional asynchrony, both measures were found to be negatively correlated (Spearman's ρ = −0.46, *p* < 4.2 × 10^−9^; Figure 1D).

**Figure 2:**
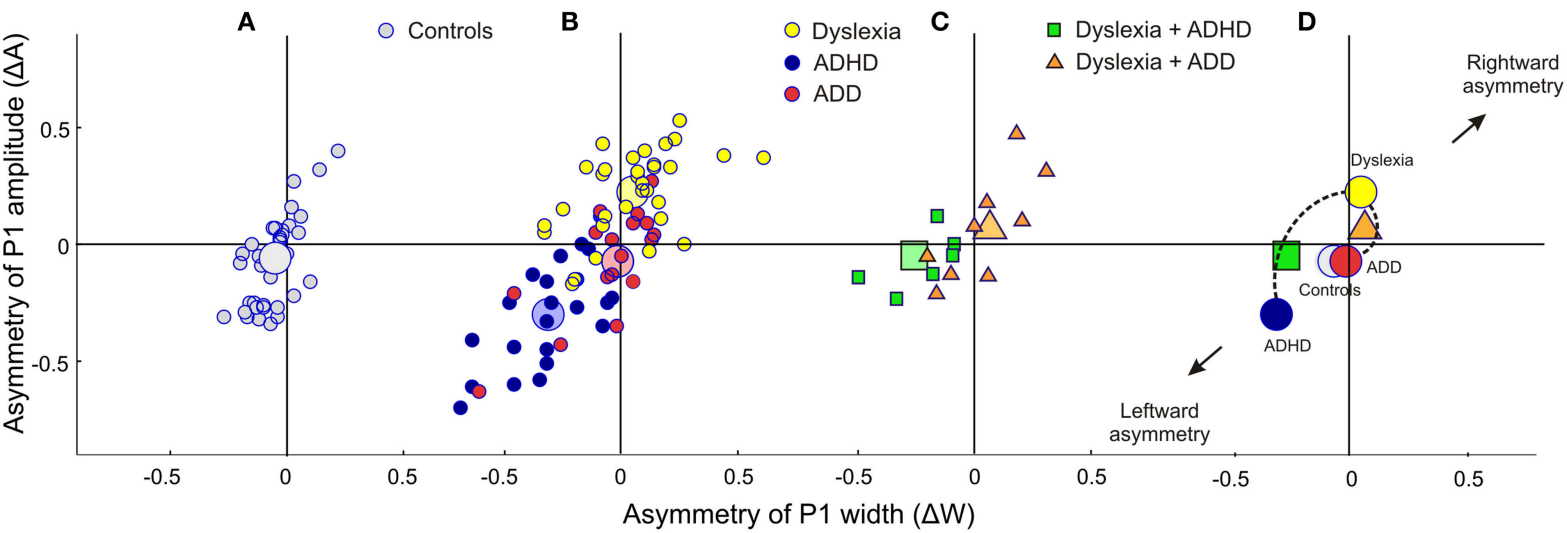
**Neurofunctional markers for a differential diagnosis of dyslexia, ADHD and ADD**. The scatterplots display group differences in relative hemispheric asymmetry Δ = (R-L)/(R+L) for the P1 amplitude (ΔA) and P1 width (ΔW). While controls (**A**, gray circles) and ADD children (**B**, red circles) show fairly symmetric patterns on both axes, the other two groups demonstrate remarkable asymmetries: dyslexics (yellow circles) are characterized by an atypical right-sided P1 amplitude enhancement, whereas ADHD subjects (blue circles) are characterized by a respective right-sided reduction in P1 amplitude and width (c.f. Table 4). **(C)** Comorbidities: apart from our large sample of 110 unambiguously assigned dyslexic, ADHD and ADD children, a further small group (*N* = 15) was diagnosed as having dyslexia combined with ADHD or ADD. **(D)**, On the average comorbid cases are located just in between the respective unequivocal groups, suggesting that comorbidities represent hybrids with regard to the considered neurofunctional parameters. Large symbols represent the centers of gravity for the respective groups.

**Figure 3:**
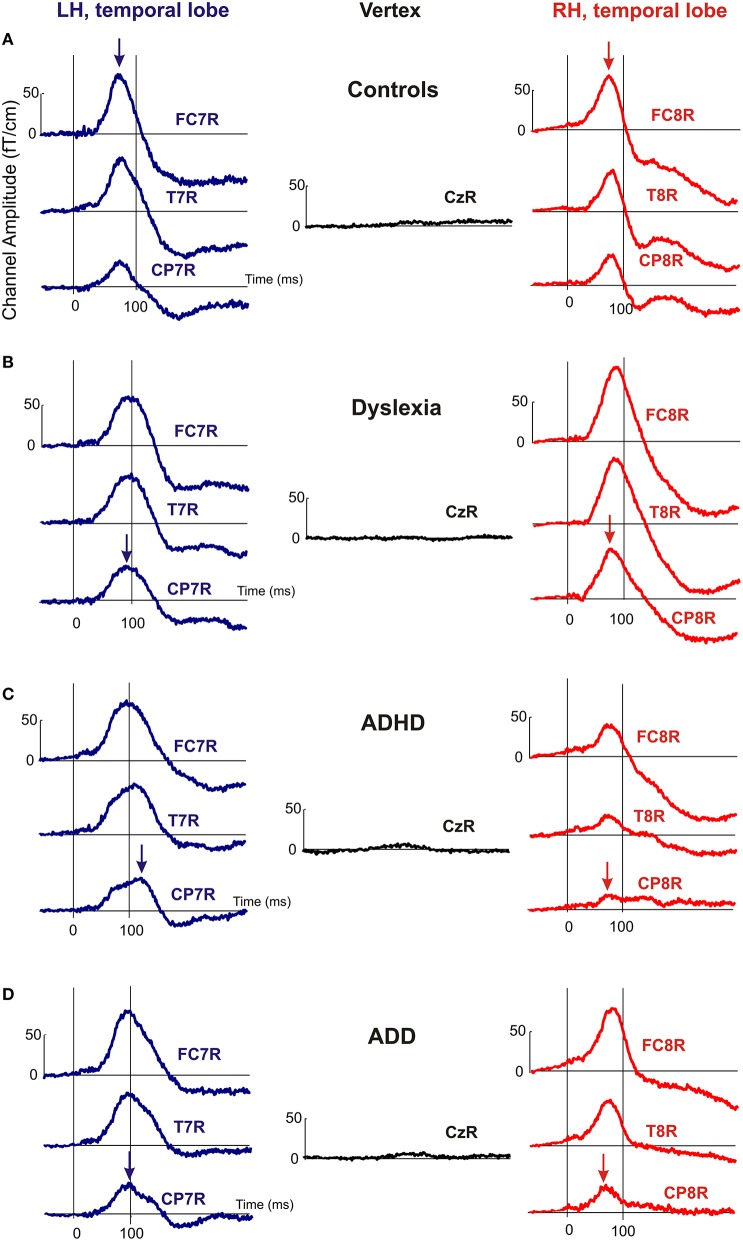
**Representative channel waveforms, group averaged over controls, dyslexics, ADHD, and ADD children**. Left hemisphere: blue curves; right hemisphere: red curves. Arrows indicate the peak positions of the P1 response, demonstrating that the characteristic left-right asynchronies obtained for the source waveforms of controls **(A)**, dyslexics **(B)**, ADHD children **(C)**, and ADD children **(D)** are also visible at the level of single sensors.

Moreover, the disorder subgroups were characterized by specific source waveform shapes. In dyslexics the P1 peak was enlarged in the right-hemisphere, while in ADHD-children the response patterns were temporally expanded in the left and diminished in the right hemisphere. ADD children showed similar, but time-shifted waveform shapes on both sides. In order to quantify interhemispheric differences in source waveform shape, relative asymmetry values were determined for P1 latency (ΔL), amplitude (ΔA), and width (ΔW) and compared across groups (see Materials and Methods, Figures 1C, 2, Table 4). Remarkably, the characteristic left-right P1 asynchronies obtained for the source waveforms are also visible at the level of representative single channel waveforms of the temporal lobe (Figure 3).

### Auditory skills

**Figure 4:**
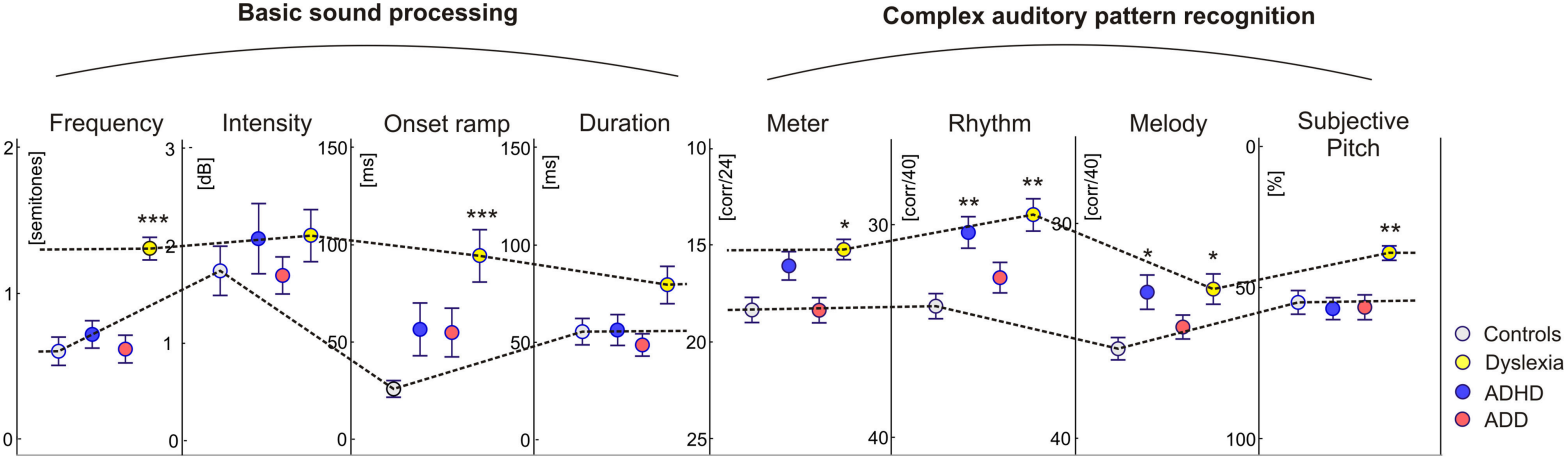
**Auditory skills**. As compared to the control group, dyslexics showed significantly poorer performance in basic hearing tasks (frequency and onset ramp discrimination) and complex sound processing (meter, rhythm, and melody differentiation). Moreover they showed a relative predominance for spectral/timbral aspects of subjective pitch perception in the Auditory Ambiguity Test (AAT). Children with ADHD were characterized by lower scores in the rhythmic and melodic subscales of the Intermediate Measures of Music Audiation (IMMA), whereas children with ADD did not show any auditory impairment at all. As all children performed normally on the intensity subtest, it is unlikely that the poorer discrimination abilities of ADHD children and dyslexics are caused by different inattention levels. Asterisks indicate significant differences between disorder subgroups and normal controls (^*^p ≤ 0.05, ^**^p ≤ 0.01; ^***^p ≤ 0.0001).

In addition to the described neurological markers, the children's behavioral performance of basic sound processing (discrimination of frequency, intensity, onset ramp, and tone duration) and more complex auditory pattern recognition, such as meter, rhythm, melody, and pitch perception, were tested (see Materials and Methods). As compared to the control group, dyslexics performed significantly poorer in almost all categories. Moreover, their subjective pitch perception was dominated by the aspect of spectral timbre. Children with ADHD were characterized only by lower scores in melodic and rhythm processing, whereas children with ADD did not show any auditory impairment at all (Figure 4).

### Beneficial influence of musical training

**Figure 5:**
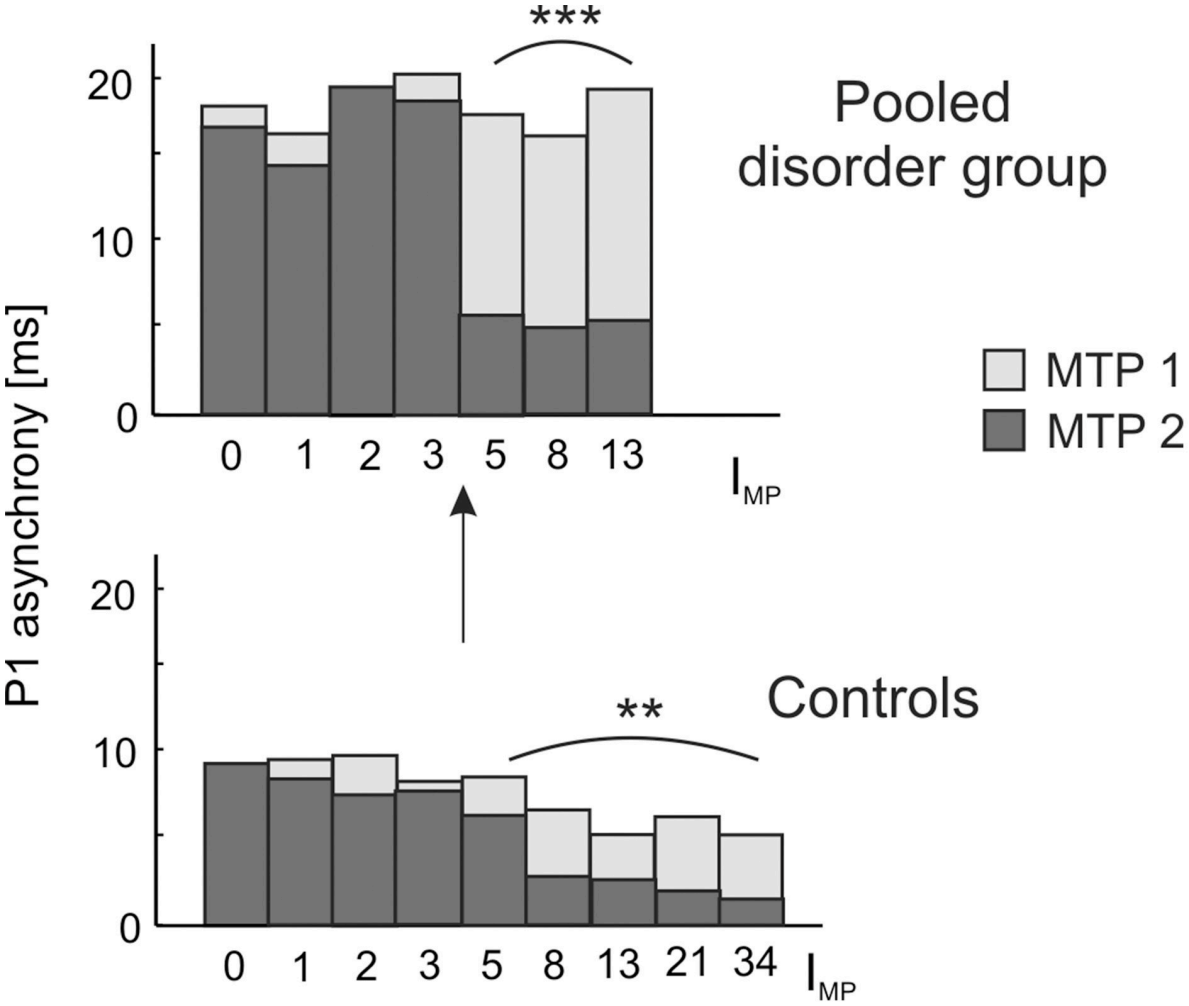
**Longitudinal development of absolute P1 asynchrony from measurement timepoint (MTP) 1 (age of 8–9 years; light bars) to MTP2 (age of 12 years; dark bars) in relation to the index of musical practice (I_**MP**_) at MTP2**. A significant correlation between I_MP_ and the degree of bi-hemispheric synchronization over time is observed as well for the pooled disorder group (Spearman's ρ = 0.58, ^***^p = 0.0004; upper panel) as for the control group (Spearman's ρ = 0.27, ^**^p = 0.009; lower panel). Both groups show a substantial increase in synchronization for I_MP_ values ≥ 5, corresponding to a minimum of e.g., 1 h of practice per week over 5 years or 5 h of practice over 1 year. Due to a more pronounced initial imbalance, musical training has a stronger effect on the synchronization of left and right AC in children with developmental disorders, thus underlining the high impact of early music-pedagogic and—therapeutic interventions on dyslexia, ADHD and ADD.

Due to our previous findings that musical training leaves the gross morphological structures of AC unchanged, but increases the neural efficiency of AC functions (Seither-Preisler et al., 2014), we addressed this question in more detail in the current study. A longitudinal analysis of auditory evoked responses in in a sub-sample of 109 children, who were still available after a time-span of 3.6 years (MTP1: age of 8–9 years; MTP2: age of 12 years; see Materials and Methods) revealed that P1 latencies were significantly shorter and bilaterally balanced in young musicians (absolute asynchrony: 12.0 ± 1.2 ms) than in non-musicians (16.6 ± 1.1 ms); c.f. Table 4. While in the longitudinal comparison the asynchrony was virtually unchanged in non-musicians, musicians exhibited a mean P1 asynchrony reduction of 5.5 ± 1.0 ms (*F*_(1, 105)_ = 47.3, *p* = 4.5 × 10^−10^, part. η^2^ = 0.31). The extent of this long-term synchronization was directly correlated to the amount of musical practice between MTP1 and MTP2 (controls: ρ = 0.27, *p* = 0.009; pooled disorder group: ρ = 0.58, *p* = 0.0004); for more details please see Figure 5. Noteworthy, the benefits of musical training were considerably larger in the pooled disorder group (14.0 ± 2.9 ms; upper panel of Figure 5) than in the control group (mus: 3.4 ± 0.6 ms; lower panel of Figure 5); *F*_(1, 105)_ = 19.1, *p* = 2.9 × 10^−5^, part. η^2^ = 0.15.

**Figure 6:**
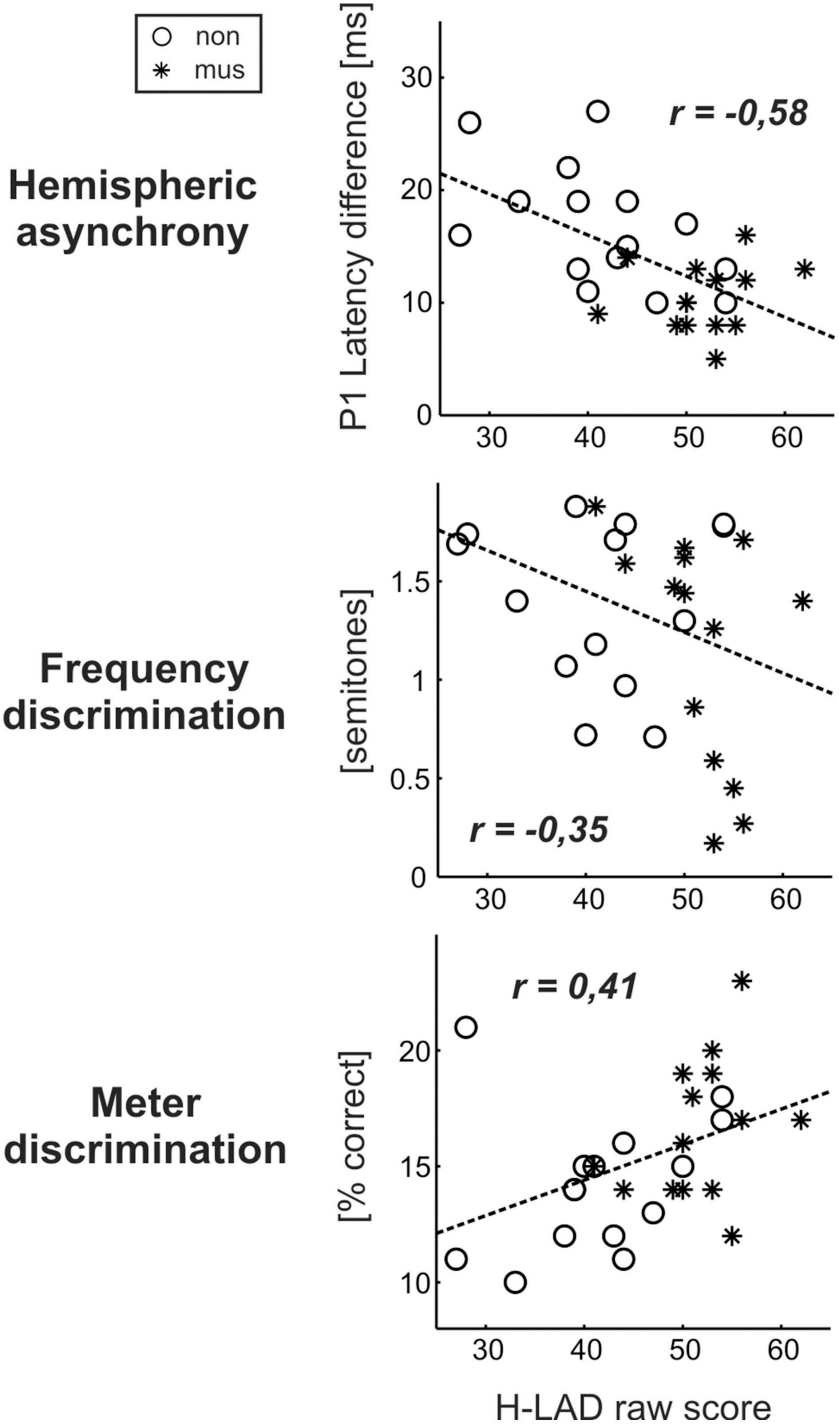
**Correlations between the dyslexics' performance in the H-LAD Test (Brunner et al., 2008), measuring correct phoneme discrimination, and parameters of the neuro-auditory profile (“musicians”: asterisks, “non-musicians”: circles)**. Good phoneme discrimination is associated with a balanced bi-hemispheric activation (low P1 asynchrony: upper panel) as well as fine frequency and meter discrimination (low just noticeable differences for frequency: middle panel; high score on Metric test: lower panel). It is evident that musically active dyslexics have advantages with regard all considered aspects of the neuro-auditory profile.

Dyslexics demonstrated the greatest variety of perceptual problems as well with regard to basic sound processing as to more complex pattern recognition (Figure 4). Furthermore, in this group the degree of hemispheric P1 asynchrony was negatively correlated to the ability to discriminate speech phonemes (*r* = −0.58, *p* = 0.0009; Figure 6, upper panel). Dyslexic musicians demonstrated a significantly higher proportion of correctly perceived phonemes (84%) than age-matched dyslexic non-musicians (66%) and performed better at the discrimination of frequency (middle panel) and meter (lower panel). These findings corroborate the general positive influence of musical practice on basic sound processing and complex auditory pattern recognition, as well as on speech processing as an important precursor of literacy (Seither-Preisler et al., 2014).

### Step-wise brain-based diagnosis of developmental disorders

#### Step 1: general identification

As a first step a discriminant analysis was performed to test how well left AC morphology (“HG/PT ratio left”) and the functional synchronization between the right and left hemisphere (“Absolute P1-asynchrony”) segregate the control group from the pooled disorder group. The established discriminant function allowed for a correct assignment of 84.4% of all cases (Table 5, analysis level 1). Both considered parameters turned out to be highly relevant for group segregation, reflecting the fact that children with dyslexia, ADHD, and ADD shared the abnormities of an oversized left-hemispheric PT and a substantial asynchrony of the primary auditory response (Figure 1D).

**Table 5:** **Results of discriminant analyses**.

**Comparison**	**Sample**	**Wilk's Lambda**	**Discrimination accuracy (Hit rate)**	**Predictors sorted by descending relevance**	**Contribution to discriminant function (r)**	**Group-specific 95% confidence intervals**
**ANALYSIS LEVEL 1: GENERAL**						
Cont vs. Dis	Orig	λ = 0.62, χ^2^ = 67.7, *df* = 2, *p* = 2 × 10^−16^	84.4%	1. P1-asynchrony [ms]	0.84	Cont: 2.5 to 4.6 Dis: 16.1 to 20.4
				2. HG/PT ratio left	−0.65	Cont: 1.9 to 3.4 Dis: 1.2 to 1.7
**ANALYSIS LEVEL 2: DIFFERENTIAL**						
Dysl vs. ADHD	Rev	λ = 0.19, χ^2^ = 62.7, *df* = 11, *p* = 2.9 × 10^−9^	96.5%	1. ΔA	0.66	Dysl: +0.16 to +0.29 ADHD: −0.37 to −0.18
				2. ΔW	0.42	Dysl: −0.03 to +0.12 ADHD: −0.38 to −0.23
	Orig	λ = 0.28, χ^2^ = 64.2, *df* = 11, *p* = 1.5 × 10^−9^	93.2%			
Dysl vs. ADD	Rev	λ = 0.28, χ^2^ = 40.9, *df* = 11, *p* = 2.5 × 10^−5^	90.7%	1. Frequency [semitones]	−0.54	Dysl: 1.1 to 1.5 ADD: 0.3 to 0.6
				2. ΔA	−0.51	Dysl: +0.16 to +0.29 ADD: −0.18 to +0.02
				3. HG/PT ratio right	0.42	Dysl: 0.84 to 1.27 ADD: 1.72 to 3.28
				4. Subjective pitch [%]	0.41	Dysl: 34.0 to 45.0 ADD: 52.1 to 75.1
				5. Meter [corr/24]	0.39	Dysl: 14.2 to 16.5 ADD: 17.7 to 21.1
				6. Rhythm [corr/40]	0.34	Dysl: 27.6 to 30.1 ADD: 31.1 to 35.7
	Orig	λ = 0.5, *χ2* = 31.8, *df* = 11, *p =* 8.2 × 10−4	79.5%			
ADHD vs. ADD	Rev	λ = 0.32, χ^2^ = 36.3, *df* = 11, *p* = 1.5 × 10^−4^	87.2%	1. ΔW	0.57	ADHD: −0.38 to −0.23 ADD: −0.15 to +0.03
				2. HG/PT ratio right	0.51	ADHD: 0.71 to 1.19 ADD: 1.72 to 3.28
				3. ΔA	0.42	ADHD: −0.37 to −0.18 ADD: −0.18 to +0.02
				4. ΔL	0.42	ADHD: −0.13 to −0.09 ADD: −0.09 to 0.00
				5. Meter [corr/24]	0.38	ADHD: 13.8 to 17.2 ADD: 17.7 to 21.1
	Orig	λ = 0.50, χ^2^ = 37.7, *df* = 11, *p* = 8.9 × 10^−5^	78.1%			

*A set of neurological markers and psychoacoustic test values was sufficient to precisely and objectively identify children with developmental disorders and/or learning deficits. Level 1: General identification [segregation of pooled disorder group (Dis) from normal controls (Cont)], Level 2: Differentiation between the disorder subgroups dyslexia (Dysl), ADHD, and ADD. The respective hit rates for an overall discriminant analysis of level 1 and 2 parameters are indicated in **Figure 8**. Contribution: correlation (r) of parameter with established discriminant function. Only parameters with contributions to the discriminant function of r ≥ |0.3|, which signify a sufficient relevance, are considered*.

#### Step 2: differential diagnosis of dyslexia, ADHD and ADD

In an attempt to discern the different types of developmental disorders, as a second step pair-wise discriminant analyses were performed on those neurological and behavioral measures that showed reliable differences between disorder subgroups (Table 5, analysis level 2). To account for the obvious fact that pediatric diagnoses may be inaccurate to some extent, we introduced additional criteria to make classifications as reliable as possible and defined a “revalidated sample” (see Materials and Methods).

##### ADHD vs. dyslexia

Segregation accuracy was 93.2% for the original sample and 96.5% for the revalidated sample. The most important factors contributing to the discrimination function were “ΔA” and “ΔW,” reflecting the fact that dyslexics were characterized by substantially larger and broader right-hemispheric P1 peaks.

##### ADD vs. dyslexia

In this case, segregation accuracy was 79.5% for the original sample and 90.7% for the revalidated sample. The most important contributing factors were “Frequency discrimination,” “ΔA,” “HG/PT ratio right,” “Subjective pitch,” “Metric score,” and “Rhythm score.” In particular, dyslexics showed poorer frequency, meter, and rhythm discrimination, a more timbre-based perception of harmonic sounds, substantially larger right-hemispheric P1 responses and — due to a larger right-hemispheric HG — a lower corresponding right HG/PT-ratio.

##### ADHD vs. ADD

For this comparison, the accuracy of group segregation was 78.1% for the original sample and 87.2% for the revalidated sample. The most relevant contributing factors were “ΔW,” “HG/PT ratio right,” “ΔA,” “ΔL,” and “Metric score.” ADD children were characterized by broader, larger, and slower right hemispheric P1 peaks, which were similar to the patterns observed in the normal control group, and — due to a larger right-hemispheric HG — a higher corresponding right HG/PT ratio. Moreover, ADHD children performed poorer at discriminating meter, rhythm, and melody (Figure 4).

**Figure 7:**
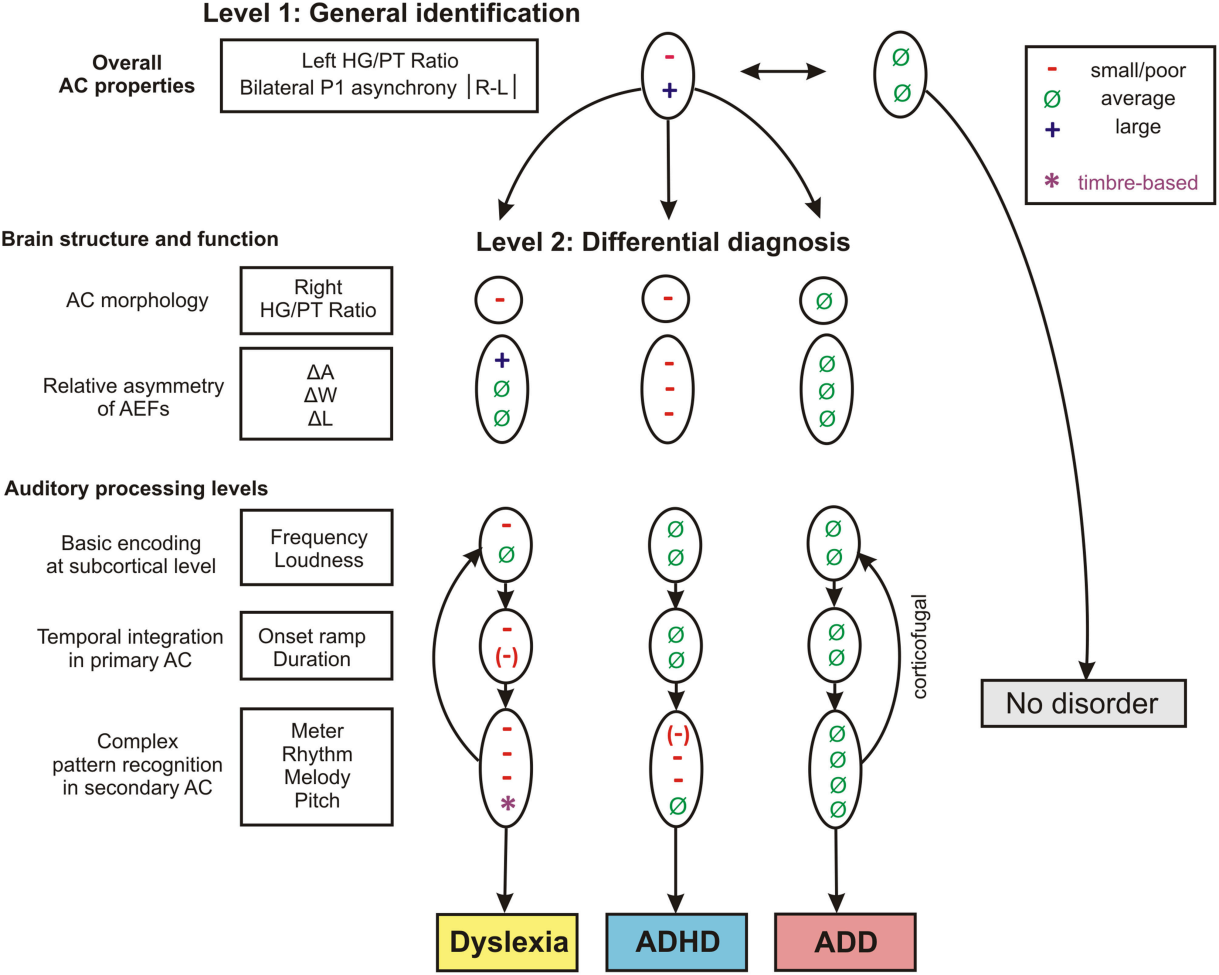
**Schematic path for a brain-based diagnosis of developmental disorders**. The two consecutive discriminant analyses steps (level 1: Segregation of pooled disorder group from normal controls, level 2: Differentiation between the disorder subgroups dyslexia, ADHD, ADD) are illustrated from top to bottom. On level 2 the indicated neuroanatomical and—functional markers are most relevant for a differential diagnosis, however auditory tests on basic sound processing and complex auditory pattern recognition further enhance diagnostic accuracy (see Table 5).

The findings of the two discriminant analysis steps and their diagnostic implications are graphically illustrated in Figure 7.

#### Diagnostic accuracy: hit rate, sensitivity, and specificity

**Figure 8:**
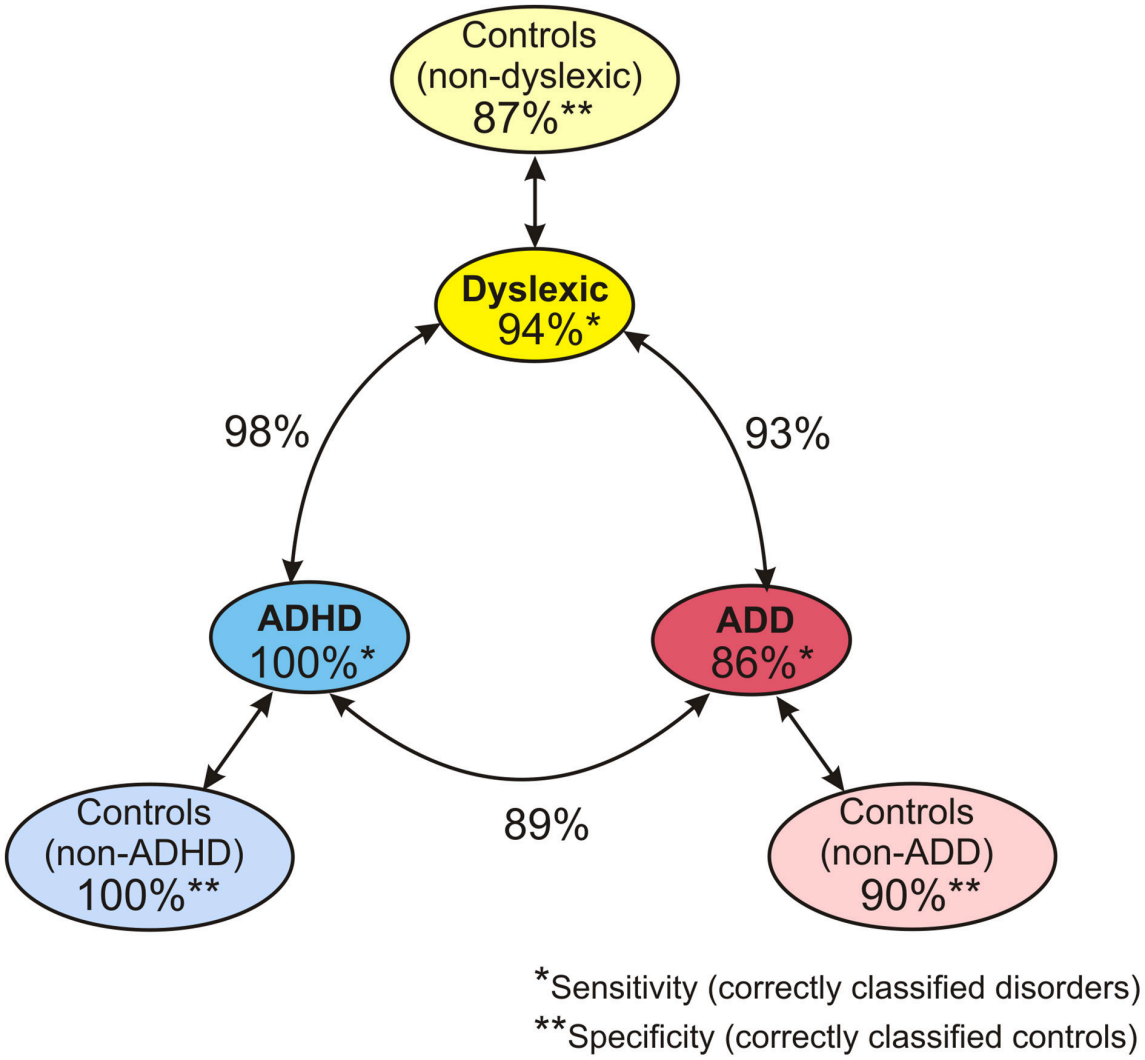
**Diagnostic validity of neuro-auditory profile**. A combination of all relevant parameters used in discriminant analysis levels 1 and 2 (revalidated sample) leads to an excellent diagnostic accuracy concerning the capacity to correctly identify cases with dyslexia, ADHD, and ADD (sensitivities; marked by ^*^), the capacity to correctly identify normal controls relative to the three disorder subtypes (specificities, marked by ^**^), and the distinction of the three disorder subtypes (hit rates indicated next to circle arrows).

When considering the parameters of the first and second analysis step together, in the original sample the three disorder groups could be discerned with the following hit rates: “ADHD vs. dyslexia”: 90.5%, “ADD vs. dyslexia”: 83.6%, “ADHD vs. ADD”: 80.8%. In the revalidated sample, these rates even increased to 98.2, 92.6, and 89.4%. Moreover, in the revalidated sample excellent sensitivity values signifying the correct identification of affected cases (ADHD: 100%, ADD: 86%, dyslexia: 94%) and specificity values signifying the correct identification of controls (non-ADHD: 100%, non-ADD: 90%, non-dyslexia: 87%) were obtained (Figure 8).

## Discussion

By combining neuroimaging and behavioral testing, we identified biomarkers for auditory-related developmental disorders. Affected children demonstrated neuroanatomical changes in left AC and a pronounced asynchrony of left and right hemispheric activation.

In the following, possible underlying mechanisms are discussed and a step-wise diagnostic procedure is suggested.

In a first step, anatomical and functional markers of AC, which had previously been observed in younger children with ADHD (Seither-Preisler et al., 2014), were similarly identified for ADD and dyslexia. These comprised an atypically enlarged left PT and a characteristic asynchrony of primary auditory responses.

Galaburda et al. (1994) first reported in a post-mortem study that dyslexics show an increased incidence of brain anomalies (ectopias, microgyri) in perisylvian areas. Later findings from molecular genetics (Paracchini et al., 2006; Wang et al., 2006) revealed that candidate genes for dyslexia are associated with neuronal migration or axon guidance. It is therefore likely that disruption of neuronal migration causes cortical anomalies of the type found in the brains of individuals with dyslexia (Hämäläinen et al., 2011). Apart from such genetic influences, from birth to puberty the overall number of cortical neurons and synapses decreases as a consequence of maturational and use-dependent plasticity (Iglesias et al., 2005). A disturbance of this process in the form of diminished or delayed pruning may result in oversized anatomical structures and functionally inefficient neural networks (Seither-Preisler et al., 2014). The morphological findings of enlarged PTs in left AC (dyslexia, ADHD, and ADD: *p* < 0.01) and to a lesser extent in right AC (dyslexia: *p* < 0.05, ADHD: *p* < 0.01) may therefore be due to genetic factors, a lack of neural pruning, or both. We speculate that these morphological anomalies may hinder the build-up of reliable interconnections between bilaterally homotopic regions via the corpus callosum (Westerhausen et al., 2009). As a compensatory reaction, alternative indirect neural connections may arise and lead to the observed atypical neurofunctional patterns in children with dyslexia, ADHD, and ADD (posterior shift of left-hemispheric P1 source and bilaterally highly asynchronous P1 latencies).

As yet, three left-hemispheric neural systems for reading have been identified, which exhibit altered levels of activation in dyslexics (Sandak et al., 2004; Shaywitz et al., 2006; Richlan et al., 2009). The dorsal posterior system is located in temporo-parietal areas (angular gyrus, supramarginal gyrus, posterior part of superior temporal gyrus/Wernicke area) and serves phonological word processing. Due to multi-sensory integration, this system is also strongly related to phoneme–grapheme conversion (Shaywitz et al., 2006). The ventral posterior system (“visual word form area”) is situated in occipito-temporal regions (left inferior occipitotemporal/fusiform area with extension into the middle and inferior temporal gyri) and is important for rapid and automatic visual word processing. It has been suggested that the dorsal posterior circuit, which is responsible for phonological processing, predominates at first and that with increasing reading skills the recognition of printed words by ventral sites becomes more central (Pugh et al., 2000). Both left-hemispheric posterior systems are typically under-activated in dyslexics (Shaywitz and Shaywitz, 2005). The third anterior (inferior frontal) system is associated with active language production, articulation, and inner rehearsal. It is located around the inferior frontal gyrus (Broca area) and tends to be over-activated in dyslexics, presumably due to compensatory strategies (Hoeft et al., 2011). Moreover, there is evidence for a right-hemispheric compensation of dysfunctional left-hemispheric posterior regions (Pugh et al., 2000; Sandak et al., 2004; Shaywitz and Shaywitz, 2005; Gebauer et al., 2012). Remarkably, the “Jyväskylä Longitudinal Study of Dyslexia” found that already a few days after birth the auditory evoked MMN in response to changes in vowel duration within consonant-vowel syllable sounds (such as /ka:/ vs. /ka/) was different for children with and without a family risk for dyslexia. Group differences emerged in terms of hemispheric preference for right hemisphere processing in the risk group vs. left hemispheric preference for the non-risk group. Furthermore, more pronounced right hemisphere processing of consonant-vowel speech sounds (e.g., /ba/, /da/, /ga/) was apparent in the newborn risk children compared to control children (for a review see Lyytinen et al., 2015). Our current finding of an enlarged right-hemispheric P1 response in dyslexics is consistent with these findings. It suggests that the right-hemispheric compensation mechanism already involves the area of the P1 source (posteromedial Heschl's gyrus), from where sensory inputs are conveyed to later stages of auditory and multi-sensory processing, including the dorsal and ventral posterior reading systems.

According to multi-time resolution models (Hickok and Poeppel, 2007; Giraud and Poeppel, 2012), oscillation-based parsing (Morillon et al., 2010; Giraud and Poeppel, 2012) and temporal sampling (Goswami, 2011; Leong and Goswami, 2014) of speech, there is evidence for a predominant lateralization of low-gamma sampling (25–40 Hz) of phonemes to the left AC and theta/delta sampling (1–7 Hz) of suprasegmental, slowly changing acoustic cues, such as syllables and phrases, to the right AC. Both processes are supported by oscillatory networks in different cortical layers of the same hemisphere that are synchronized in a nested fashion (Giraud and Poeppel, 2012). Based on our current findings we propose an extended two-step model with a first intra-hemispheric and a second inter-hemispheric analysis stage that provide an optimal representation of different timescales in speech and music. Efficient first level processing should be reflected in a fast and sharp unilateral response with strong phase coupling between different cortical layers. In contrast, a slow, shallow or multi-peaked waveform, which can be frequently observed in children with developmental disorders, should characterize an immature neurological state that produces a temporally disintegrated output (Sharma et al., 1997). Such unilateral imprecision, in turn, should impair second level bilateral integration. Furthermore, spatial asymmetries in AC activation, as evident from the left-hemispheric shift of the P1 source from HG toward PT, may be problematic, since the bilateral functional mapping is no longer homotopic (Westerhausen et al., 2009). This may impair the transcallosal exchange of information and the integrative processing of fast and slowly changing acoustic features. As yet, multi-time resolution models have been primarily discussed in the context of speech processing and literacy, with evidence for atypical activation patterns in dyslexia (Goswami, 2011; Leong and Goswami, 2014). The consistent finding of an altered left-hemispheric AC morphology together with bilateral P1 asynchrony across the three studied disorder subgroups suggests that such models may be relevant for attentional functions and the etiology of AD(H)D, as well. Our results suggest a bottom-up explanation of the interrelationship between auditory and attentional dysfunctions, where inefficient auditory processing has a negative effect on sustained attention and working memory, since more resources are captured for low-level signal analysis.

Although, the neurological findings described so far clearly indicate that AC anomalies are involved in each of the disorder subgroups, they do not explain, why auditory performance was different. Dyslexics revealed a wide range of impairments, while children with ADHD had more specific problems and children with ADD did not show any impairment at all. Hence, in a second step additional parameters were considered to allow a more reliable differentiation of subgroups.

Previous studies have reported a variety of auditory deficits in dyslexics (Hornickel and Kraus, 2013; Hämäläinen et al., 2013; Lehongre et al., 2013) that constrain the development of phonological representations and hence of literacy skills (Seither-Preisler et al., 2014; Hämäläinen et al., 2015). In our current study deficits were observed as well for basic sound features as for more complex auditory patterns. Basic auditory discrimination functions already origin from the brainstem (Wong et al., 2007; Moerel et al., 2015). In dyslexics impoverished neural sound representations have been found for subcortical, cortical, and corticofugal functions (Song et al., 2008; Lehongre et al., 2013) that are associated with an oversampling and decoding of subphonemic information especially in left AC (Giraud and Ramus, 2013). This should not only reduce sensitivity to onset ramps, formant transitions and voicing in speech sounds (Hämäläinen et al., 2012), but also cause an overflow of auditory working memory that contributes to attentional problems (Seither-Preisler et al., 2014). Moreover, the right AC exhibits reduced theta and delta oscillations (Abrams et al., 2008; Goswami, 2011; Hämäläinen et al., 2012). We propose that such alterations are eventually reflected in a functional disintegration of left and right AC, which impairs the build-up of increasingly complex information chunks (phonemes, phrases, rhythms, melodies) and leads to poor phonological awareness, verbal short-term memory, and slow performance in rapid automatized naming tasks. The strong negative correlation between P1 asynchrony and phoneme discrimination supports this view. Morphologically, such anomalies are paralleled with a high occurrence of complete posterior right HG duplications, which in this study are considered as a part of PT (Altarelli et al., 2014). As right AC subserves spectral pitch perception of speech vowels and musical sounds (Schneider et al., 2005), this may also explain the more timbre-based auditory perception of dyslexics.

The ADHD group only showed substantial higher-order deficits in the perception of rhythm and melody. In view of the multimodal organization of AC (Scheich et al., 2011) it appears likely that the observed auditory deficits also affect higher cognitive functions. From that perspective, comorbidities between dyslexia and ADHD are — at least in part — due to common problems in temporal pattern recognition and auditory attention that are mediated by coherent cortical networks. The primary auditory responses of ADHD children were delayed on the left side and diminished, but sharpened, on the right side. Future research will have to clarify, whether this specific pattern reflects a right-hemispheric deficit or compensation mechanism.

Unlike the other disorder subgroups, children with ADD showed good performance in all auditory tests and exhibited a normal-sized right HG. This suggests that in ADD left-hemispheric anomalies may be efficiently compensated by the well-developed right HG. Furthermore, the bilateral source waveforms, though being shifted in latency, were fairly balanced in shape. This may be a sign that functional AC maturation is not critically affected, but just delayed, which is reflected by a developmental lead of the earlier maturing right hemisphere. Neuroanatomical studies in fetuses have shown that the development of HG is established during the 31st week of gestational age. In most cases, right HG develops 1–2 weeks earlier than the left (Chi et al., 1977). Consistently, during the first months of life the right superior temporal regions mature relatively faster (Leroy et al., 2015). If the time-shifted source waveforms in ADD are indeed a sign of a somewhat slower but functionally efficient development of AC, this raises the important question, whether the attentional problems in ADD are different from those in ADHD, a topic repeatedly addressed in the historical controversy about the inclusion of ADD into the DSM-III-R and DSM-IV manuals (Nadeau, 1995).

In a diffusion tensor imaging study, Lei et al. (2014) found first evidence for differential cortical wiring pathways in AD(H)D subtypes. Both ADD and ADHD were associated with abnormalities in temporo-occipital areas (left middle and superior temporal gyri and left occipital lobe/cuneus). Wang et al. (2013) reported a significant positive correlation of resting state activity in the bilateral cuneus and precuneus with inattentive scores. This suggests that the observed changes are related to inattention, which is a common characteristic of ADD and ADHD. In ADHD, additional abnormalities were found in the frontal lobe, including both motor and behaviorally relevant circuits. In particular, frontostriatal and fronto-subthalamic circuits, which are thought to involve response inhibition and executive control, were affected. So while inattention appears to be related to a stronger involvement of the posterior default network, a neurological state characteristic for day-dreaming, hyperactivity and impulsivity appear to be linked to a decrease in frontal executive control.

There is growing evidence for a close relationship between divergent thinking, which corresponds to the idea finding phase in a creative process, and default network activation (Beaty et al., 2014). As our current findings demonstrate that children with ADD show a higher musicality than children with ADHD or dyslexia, it may be speculated, if ADD is associated with a higher creative potential that distracts attention from external tasks to inner imagination and audiation (Gordon, 1986). Comparatively, children with ADHD appear to face more basic problems that are linked to neurological anomalies as well in secondary AC as in prefrontal cortical networks relevant for executive control (Konrad and Eickhoff, 2010). This would be consistent with the assumption of differential cortical wiring pathways in ADHD and ADD, as reflected in the strikingly different source waveform shapes. The ADHD-specific pathway may involve an over-activation of the externally directed orienting network responding to novel stimuli (Raz and Buhle, 2006) and thereby distracting attention from intended tasks (van Mourik et al., 2007), whereas the ADD-specific pathway may predominantly involve an over-activated default network that supports internally directed imagination, audiation, and cognition (Spreng et al., 2013).

Two-step discriminant analyses revealed that a set of neurological markers and psychoacoustic test values was sufficient to precisely and objectively identify children with developmental disorders. The first step aimed to identify the pooled disorder group on the basis of common left-hemispheric anomalies in AC morphology and functional asynchrony. By additionally considering the individual characteristics of right AC and hearing performance, it was possible to differentiate between the three disorder subgroups with outstanding accuracies of 89–98%. This by far exceeds the diagnostic validities of most questionnaires and psychological tests in this field (Tripp et al., 2006; Snyder et al., 2008, 2015).

We thus believe that our innovative approach has the potential to substantially change the way auditory-related developmental disorders and learning deficits can be diagnosed. Particularly, our method may essentially contribute to the clarification of the relation between ADHD and ADD, two often confounded subtypes of attentional disorders (Chermak et al., 1999). The vast majority (~95%) of our tested AD(H)D children were under medication (mostly methylphenidate), regardless of whether they had been diagnosed to have ADD (F 98.8) or ADHD (F 90.0/F90.1) according to the ICD-10 criteria. In view of the recent evidence for differential cortical wiring pathways in ADD and ADHD, with the latter showing more widespread and severe cortical anomalies (Lei et al., 2014), it appears questionable to treat both subtypes in the same way. Our current results corroborate this view, as they demonstrate largely different perceptual and neurofunctional patterns in ADD and ADHD, with the former being more similar to normal controls. Our method may also help to identify comorbid cases, with significant consequences for the choice of appropriate therapeutic interventions by practitioners.

By applying a longitudinal approach we found clear evidence that musical training promotes interhemispheric synchronization. Regularly playing a musical instrument normalized the temporally disintegrated activation of left and right AC, which was characteristic for children with dyslexia, ADHD, and ADD at the beginning of the study. Thus, musical interventions appear to directly counteract these developmental disorders and learning deficits on a neurological level. Unlike for AD(H)D treatments with methylphenidate and related stimulants, which are controversially discussed in the public, only positive side-effects are to be expected from musical training (Schellenberg, 2004; Hyde et al., 2009; Moreno et al., 2009; Kraus and Chandrasekaran, 2010; Seither-Preisler et al., 2014; Flaugnacco et al., 2015; Tierney et al., 2015). At present, for children with dyslexia, ADHD, and ADD musical education is the exception rather than the rule. According to our observations, the majority of dyslexics with musical experience (64%) showed a preference for playing the piano (poor frequency discrimination compensated by fixed keys), while the majority of ADHD children with musical experience (59%) favored percussive instruments such as drums or guitar (expression of impulsivity). Dyslexics should benefit from playful programs that train basic tonal and rhythmic skills without score reading. Children with ADHD might take more advantage of rhythmic and melodic tasks that motivate to move freely. Rhythmic feeling, dancing and emotional expression of the whole body may enhance bi-hemispheric synchronization and entrain multisensory and coordinative skills, audio-motor coupling and attentional functions (Altenmüller et al., 2009; Tierney et al., 2015).

As a future perspective, a cross-validation of our findings may lead to an even more generalized view of the presented inferences. This would have far-reaching implications for research and practice and provide a deeper understanding of the etiology, diagnosis, and musically based therapy of common auditory-related developmental disorders.

## Author contributions

BS, CG, PS, and ASP substantially contributed to the conception and design of the work, BS, CG, DE, NG, PS, and ASP were involved in the acquisition of the data, BS, CG, VB, JB, DE, MBL, MW, MBR, AS, RP, SS, PS, and ASP in the anaylsis and interpretation of the data. BS, CG, PS, and ASP drafted the work, VB, DE, JB, NG, MBL, MW, MBR, AS, SS, and RP revised it critically for important intellectual content. All authors agree to be accountable for the content of the work and agreed to be accountable for all aspects of the work in ensuring that questions related to the accuracy or integrity of any part of the work are appropriately investigated and resolved.

### Conflict of interest statement

The authors declare that the research was conducted in the absence of any commercial or financial relationships that could be construed as a potential conflict of interest.
